# Complete Sequence, Multichromosomal Architecture and Transcriptome Analysis of the *Solanum tuberosum* Mitochondrial Genome

**DOI:** 10.3390/ijms20194788

**Published:** 2019-09-26

**Authors:** Jean-Stéphane Varré, Nunzio D’Agostino, Pascal Touzet, Sophie Gallina, Rachele Tamburino, Concita Cantarella, Elodie Ubrig, Teodoro Cardi, Laurence Drouard, José Manuel Gualberto, Nunzia Scotti

**Affiliations:** 1 Univ. Lille, CNRS, Centrale Lille, UMR 9189—CRIStAL—Centre de Recherche en Informatique Signal et Automatique de Lille, F-59000 Lille, France; jean-stephane.varre@univ-lille.fr; 2 CREA Research Centre for Vegetable and Ornamental Crops, 84098 Pontecagnano Faiano, SA, Italyconcita.cantarella@gmail.com (C.C.); teodoro.cardi@crea.gov.it (T.C.); 3 Univ. Lille, CNRS, UMR 8198—Evo-Eco-Paleo, F-59000 Lille, France; pascal.touzet@univ-lille.fr (P.T.); sophie.gallina@univ-lille.fr (S.G.); 4 CNR-IBBR, National Research Council of Italy, Institute of Biosciences and BioResources, 80055 Portici, NA, Italy; rachele.tamburino@gmail.com; 5 Institut de Biologie Moléculaire des Plantes-CNRS, Université de Strasbourg, 67084 Strasbourg, France; elodie.ubrig@ibmp-cnrs.unistra.fr (E.U.); laurence.drouard@ibmp-cnrs.unistra.fr (L.D.)

**Keywords:** mitochondria, potato, Solanaceae family, mtDNA, multichromosomal structure, repeated sequences, RNA editing, comparative genomics

## Abstract

Mitochondrial genomes (mitogenomes) in higher plants can induce cytoplasmic male sterility and be somehow involved in nuclear-cytoplasmic interactions affecting plant growth and agronomic performance. They are larger and more complex than in other eukaryotes, due to their recombinogenic nature. For most plants, the mitochondrial DNA (mtDNA) can be represented as a single circular chromosome, the so-called master molecule, which includes repeated sequences that recombine frequently, generating sub-genomic molecules in various proportions. Based on the relevance of the potato crop worldwide, herewith we report the complete mtDNA sequence of two *S. tuberosum* cultivars, namely Cicero and Désirée, and a comprehensive study of its expression, based on high-coverage RNA sequencing data. We found that the potato mitogenome has a multi-partite architecture, divided in at least three independent molecules that according to our data should behave as autonomous chromosomes. Inter-cultivar variability was null, while comparative analyses with other species of the Solanaceae family allowed the investigation of the evolutionary history of their mitogenomes. The RNA-seq data revealed peculiarities in transcriptional and post-transcriptional processing of mRNAs. These included co-transcription of genes with open reading frames that are probably expressed, methylation of an rRNA at a position that should impact translation efficiency and extensive RNA editing, with a high proportion of partial editing implying frequent mis-targeting by the editing machinery.

## 1. Introduction

Mitochondrion is a semi-autonomous organelle that supplies cells with ATP through oxidative phosphorylation. In higher plants, the mitochondrial DNA (mtDNA) displays unique features when compared with animal and fungi counterparts. It is larger and highly variable in size, ranging between 208 kb in *Brassica hirta* [1] and 11.3 Mb in *Silene conica* [2]. The mitochondrial genome (mitogenome) also varies greatly in size across species belonging to the same family, such as in Cucurbitaceae, where the mtDNA of *Citrullus lanatus* is 379 kb [3], while in *Cucumis melo* it is 2740 kb in size [4]. However, the extreme size variability is not reflected in the gene content, which remains quite constant among species and includes less than 50 protein-coding genes, mainly components of the electron transport chain, and a few rRNA and tRNA genes, accounting for about 10% of the mitogenome [5]. Most of the remaining DNA consists of non-coding sequences commonly of unknown origin, but also acquired from nuclear, chloroplast or viral DNA by horizontal transfer [6].

The plant mitogenomes are conventionally described as circular structures, and in most species they could be mapped into a single circular chromosome often called a “master circle”. However, in vivo observation often failed to recover circular molecules [7,8,9]. Rather, most studies demonstrated that the mitochondrial genome structure is more complex and dynamic, being a mixture of inter-convertible linear and circular DNA molecules that result from homologous recombination events involving large, intermediate-size (ISR) and small repeated sequences scattered throughout the genome [10,11,12]. The relative ratio of alternative structures depends on the balance between their recombination and replication rates. Specifically, the recombination events that involve ISRs are often asymmetrical and have been linked to intra-specific mtDNA variation [13]. The single circle map of the genome is also a concept that had to be revised, because of the recent identification of mitogenomes with multiple molecules, constituted by assembled identities autonomous from each other, with no evidence that they can be inter-convertible to a single molecule by recombination [11,12,14,15,16]. An extreme example was found in certain *Silene* species, which can have more than 50 autonomous molecules [2], and that evolve fast by gain or loss of entire molecules [17].

Furthermore, mitochondria of many plant species also contain circular and linear plasmid DNA molecules that exist as standalone extra-chromosomal elements that can range from 0.7 to over 20 kb [10,18,19]. Multimeric forms of these molecules, likely resulting from rolling-circle replication processes have been observed [20]. The pattern of mitochondrial plasmids can be species-specific and contribute to the complexity of mitochondrial genetics. Most of them do not show sequence similarity with the main chromosome, and thus cannot be considered as sub-genomes [10,18]. Despite the high variability in size and structure, in most plant species, the mtDNA displays a slow sequence evolution rate likely due to efficient DNA repair systems, most probably by copy-correction by recombination [21,22]. Finally, recombination events may lead to the generation of new chimeric open reading frames (orfs) that, in several cases, have been associated with cytoplasmic male sterility (CMS) [22]. CMS is a maternally inherited inability to produce or shed functional pollen, an economically important trait that can be exploited in plant breeding for the production of hybrid seeds [23,24,25].

A particularity of plant mitochondrial gene expression is extensive RNA editing by C to U deamination, required for the correct expression of its genes [26]. In all flowering plants, RNA editing is an essential step of mitochondrial RNA maturation without which no functional mitochondria can be assembled and maintained in the cell [27]. Numerous studies on RNA editing have been carried out, but how a functional editosome is assembled in plant organelles has remained elusive [28,29]. Very recently, Oldenkott et al. [30] demonstrated that single Pentatrico Peptide Repeat (PPR) proteins with a terminal DYW domain from *Physcomitrella patens* can edit their corresponding target in *Escherichia coli*. Although new sequence approaches allowed the sequencing of the mitogenomes from many plant species, the complete profile of mitochondrial editing sites has only been determined for relatively few species. Comparison between species allows good prediction of sites required for correct protein expression, but the in silico predictions are blind to editing sites that are partially edited or that result in silent substitutions.

Potato (*Solanum tuberosum* L.) is the third most important food crop in the world grown for human consumption (http://www.fao.org/faostat/en). It originates from Southern America and is nowadays cultivated in all continents except Antarctica, with China being the greatest producer (http://www.fao.org/faostat/en). The key event in potato domestication was adaptation to a long-day photoperiod, enabling global cultivation at non-equatorial latitudes [31]. Domestication also selected for enlarged tubers with a reduced content in toxic glycoalkaloids and reduced sexual fertility [31]. The potato nuclear sequence was published in 2011 [32] and since then numerous genomic analyses have been performed to characterize nuclear genome composition, to assess chromosome structure organization as well as the genetic variability of potato germplasm collections [33,34,35,36,37,38]. Furthermore, several studies have included comparisons among closely related species aimed at uncovering the evolutionary history of the Solanaceae family. Intra- and inter-specific variation in mtDNA organization was highlighted in common potato and related species [39,40,41,42], showing co-evolution patterns of the chondriome and the other cellular genomes. In this regard, studies on the mitogenome composition, structure and organization may help elucidate the genetic diversity in potato and reveal molecular mechanisms underlying nuclear-cytoplasmic interactions responsible for male fertility and expression of other agronomic relevant traits [41,43].

Herein we report the complete mtDNA sequence of two *S. tuberosum* cultivars, namely Cicero and Désirée. From PacBio RS long-reads we found that the potato mitogenome is divided in a minimum of three autonomous molecules that according to our data should behave as autonomous chromosomes, and that there is no variability between the two potato cultivars investigated. Comparative analyses with available mitogenomes for species belonging to the Solanaceae family allowed us to identify syntenic blocks and investigate the evolutionary history of its mitogenomes. Finally, from RNA sequencing data, we identified putative promoters and transcription processing sites, and characterized the extensive RNA editing pattern of potato mitochondria.

## 2. Results and Discussion

### 2.1. S. tuberosum Mitochondrial Genome Assembly

**Figure 1:**
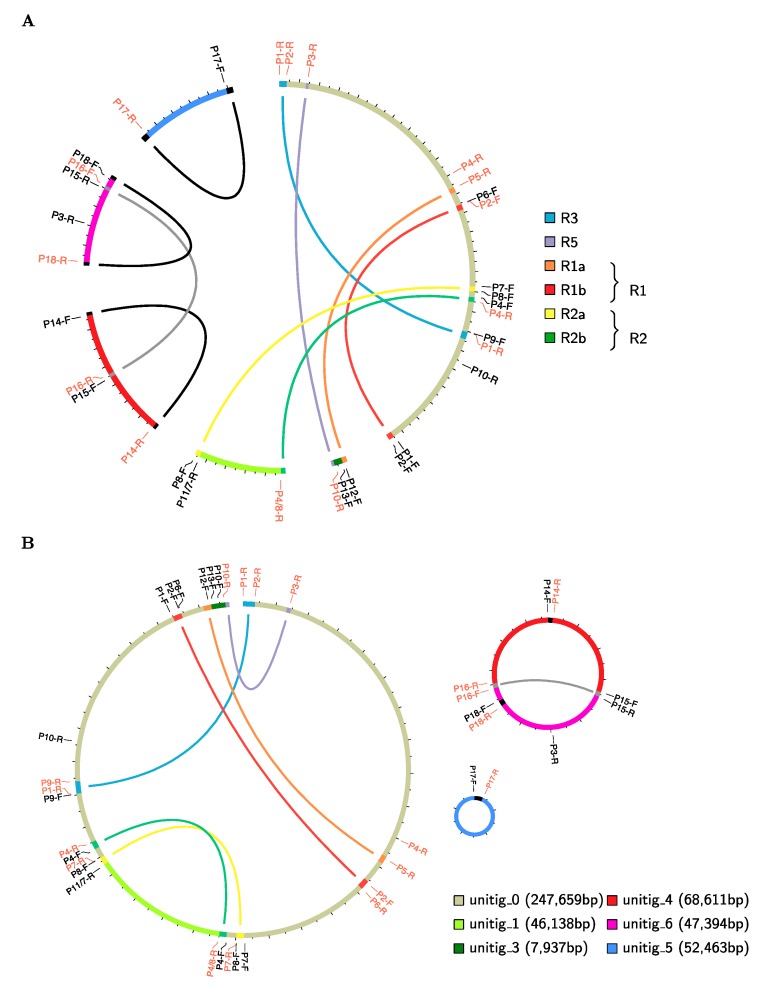
*S. tuberosum* mitochondrial genome assembly. (**A**) The 6 unitigs output by HGAP for the Cicero mitogenome. Colored blocks on the unitigs show repeats. Labeled ticks give primers positions, with those in black in forward orientation and those in light red in reverse orientation. (**B**) The 3 contigs obtained after PCR validation. Colored blocks on the contigs show repeats. The larger contig could not be circularized.

The *S. tuberosum* cv. Cicero was assembled into 6 linear unitigs (Table S1), with a total size of 470 kb. The S. *tuberosum* cv. Désirée was assembled into 5 linear scaffolds, with a total size of 475 kb (Table S1). The extraction of DNAs from purified and DNase treated mitochondria should avoid the inclusion of any NUMTs in the final assemblies. Despite the fact that the two assemblies originated from different data sets and by the application of different bioinformatic tools, they are almost identical in the primary sequence (99.99% identity), as well as in the multi-partite architecture. Therefore, hereinafter we will refer to a single assembly, namely that of *S. tuberosum* cv. Cicero (Figure 1A).

Genome assembly was complicated by the presence of direct or inverted repeats of large/medium size (colored blocks in Figure 1A, Table S2) responsible for the multi-partite configuration of the mitogenome. We initially found 23 pairs of repeats, 10 being larger than 1,000 bp. Several repeats were located at the ends of raw contigs (Figure 1A), indicating that they might exist as independent sub-genomes in vivo. After manual scaffolding the number of repeats larger than 100 bp was reduced to 18, ranging from 111 to 11,915 bp in length, (Figure 1, Table S2). The assembly graph provided by Canu gave us insights into putative scaffolding scenarios (Figure S1). By mapping repeats on reads in this graph, we were able to explore which rearrangements had to be considered. PCR experiments designed to verify alternative configurations (Table S3, Figure S2) allowed us to propose the best assembly, and to recover missing sequences filtered out during the assembly process.

We found that the potato mitogenome can be assembled into two autonomous circular molecules of 49,229 bp and 112,797 bp, plus a third linear sequence of 312,491 bp, for a final genome size of 474,520 bp (Figure 1B). The same organization was inferred to both Cicero and Désirée cultivars. Other conformations are possible, and alternative circular rearrangements can be described for the linear sequence (Figure S3). One possible configuration uses repeat R3 (blue repeat in Figure 1) leading to a circular sequence of 229,571 bp by removing the end of unitig 0 (Figure S3A). A second alternative configuration uses repeat R5 (purple repeat in Figure 1) leading to a slightly different circular sequence of 296,789 bp, by removing the beginning of unitig 0 (Figure S3B). Thus, it is possible that in vivo there is co-existence of two alternative circular forms, and no linear molecules. However, the existence of the two is required, because essential genes are present in the small regions differentiating the two alternative circular forms. PCR experiments confirmed the existence of these alternative configurations.

### 2.2. Gene Content of the Potato mtDNA

**Figure 2:**
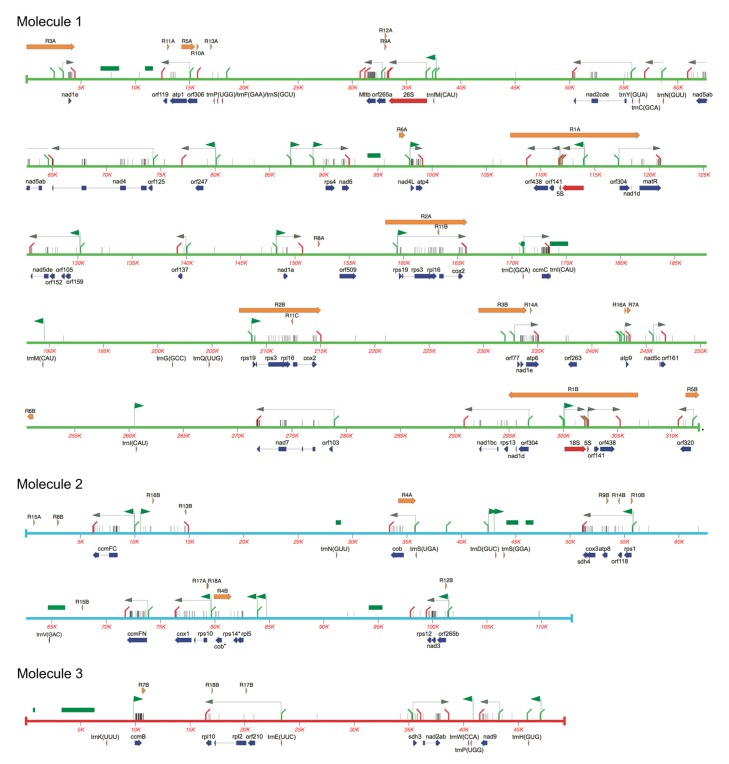
Organization of the potato mtDNA. Molecules 1, 2 and 3 are represented in green, light blue and red respectively. Gene sequences are shown below the sequence line, with protein genes in dark blue and rRNA and tRNA genes in red. Repeated sequences larger than 100 bp are shown above the sequence as orange arrows. Green bars indicate sequences of plastidial origin. Bent green and red lines indicate 5′ and 3′ transcript boundaries, respectively. Grey horizontal arrows represent major transcripts. Green horizontal arrows indicate consensus promoters found upstream of transcripts. Thin vertical lines indicate editing sites.

The analysis of the *S. tuberosum* mtDNA sequence revealed a standard set of protein-coding genes usually found in the mitogenomes of other dicot species (Table 1, Figure 2). An *rps14*-related sequence located downstream *rpl5* is a pseudogene that contains frame-shifts resulting in in-frame stop codons. This pseudogene was previously described both in potato and Arabidopsis [44,45,46], and it has been shown that a functional copy of the gene has been transferred to the nucleus [47]. This transfer occurred roughly 80 million years ago, and it is surprising that the sequence of the mitochondrial pseudogene has remained virtually unchanged [48].

The *rpl5-ψrps14* sequences were located upstream of a truncated copy of *cob* (i.e., *ψcob*), contained in the R4B repeat, and *rps10-cox1* genes (Figure 2). An alternative arrangement with *rpl5-ψrps14* in front of *cob* was reported by [45], and its occurrence in common potato and other species was later investigated [42,49]. Both arrangements, theoretically possible in *S. tuberosum* due to recombination between the R4 repeats were confirmed by PCR experiments and analysis of long reads spanning the R4 sequence.

As with all other higher plant mitogenomes, that of the potato also codes for the ribosomal RNAs 5S, 18S and 26S. A limited set of 23 tRNA genes, corresponding to 15 amino acids, was found in the genome (Table 2). Based on sequence similarity it can be inferred that nine are of plastidial origin, from the promiscuous import and insertion in the mtDNA of plastidial genomic sequences. A comprehensive two-dimensional polyacrylamide gel study of potato mitochondrial tRNAs showed that six of them could be detected in mitochondria [50]. However, sequences for trnC(GCA), trnV(GAC) and trnI(CAU) of plastidial origin are present in the genome (Table 2). By northern blot hybridization, we confirmed for the first two that the corresponding tRNAs cannot be detected in purified mitochondrial RNA and are apparently pseudogenes (Figure S4). The missing tRNA species are likely imported from the cytosol into mitochondria [50].

### 2.3. Transcriptome of the Potato mtDNA

Many orfs larger than 100 codons were found in the mitogenome. Among these, several are closely associated with known mitochondrial genes that could be potentially co-transcribed and expressed. It is also known that extensive RNA editing alters the coding sequences of most mitochondrial genes, including the creation of initiation and stop codons. Thus, a comprehensive study of the mtDNA genes expression requires transcriptomic studies that gives information on the sequences that are transcribed, potential promoters, transcript processing sites and editing. We have therefore sequenced three independent Illumina RNA-seq libraries (2 × 100 bp paired-end). Respectively, 61, 29 and 39 million reads could be aligned to the potato mtDNA. The very high coverage of the transcribed sequences allowed us to identify with precision the boundaries of most expressed transcripts and all editing sites, including those that are edited with low efficiency and those found in non-coding sequences that are transcribed at background level.

Based on the RNA-seq data, we were able to define 33 transcription units containing protein-coding genes and orfs (Table 1). Many of them contained co-transcribed genes, such has the one that includes co-transcribed genes *rps1*, *atp8*, *cox3* and *sdh4*, while others are mono-cistronic, like in the case of the transcripts of genes *cob*, *atp6* and *atp9*. Quite surprisingly, a highly expressed transcript (coordinates 56998–58596 in the (–) strand of molecule 1) does not contain any putative coding sequence. An analysis of its sequence did not reveal any particular putative secondary structure, but this long non-coding RNA (lncRNA) might assume regulatory functions in mtDNA maintenance or expression, as described for the mitochondria of several other non-plant systems [51].

Several unidentified orfs are transcribed. Among them, a few are most likely expressed, because their retention in the transcription unit does not seem fortuitous. That is the case of *orf247* and *orf137*, which are individually transcribed in their own specific transcripts (Figure S5A). An ortholog of *orf137* was previously described as potentially being involved in the CMS of chili pepper [52]. Other transcribed orfs deserve further study, as is the case of *orf265b*, which is co-transcribed with *nad3-rps12* genes (Figure S5B) and contains a characteristic ELF-domain (Pfam accession PF03317). Several of the transcribed orfs have sequence similarities to RNA-dependent RNA polymerases (RdRp) and might be of retroviral origin.

Based on RNA-seq data, it was possible to define RNA boundaries with relatively good precision. Genome annotation indicates the borders of the regions that are covered, and the true RNA boundaries should be a few additional nucleotides upstream and downstream of 5’ and 3’ ends, respectively. In the mitochondria of dicots, poorly conserved core promoter sequences have been described, with primary transcripts initiating a few nucleotides downstream of a CRTA conserved element, or of TATA-like sequences, such as TATTA [53,54,55]. We looked for putative promoters upstream of the 5’ ends of transcripts. Eleven putative promoters were so identified, containing CRTA or TATATAA core elements (Table S4). The other 5’ transcript ends that were identified are probably processing sites.

The presence of RNA structures that could be involved in transcript processing and or stability was also investigated. It is known that tRNA sequences and tRNA-like RNA structures (t-elements) that are processed by RNases P and Z are often used as processing sites for protein-coding transcripts. In potato mitochondria we found that processing of five tRNAs by RNase Z is used as a signal to define the 5’-ends of as many transcripts (rRNA *26S* precursor, *nad2cde*, the lncRNA, *cob*, and *ccmC*) (Table 3). A particular case is the 3’ processing of the *ccmC* transcript. A tRNACys gene of plastidial origin overlaps the C-terminus of the *ccmC* orf. The processing of this tRNA sequence by RNase P results in a truncated transcript of 92 nucleotides with no stop codon. The processing of this tRNA suggests that it is a functional tRNA potentially involved in translation. However, as described above, it has not been detected in the total population of potato mitochondrial tRNAs [50]. Northern blot analysis (Figure S4) shows a very weak signal in mitochondria as compared with chloroplasts. This signal is likely due to plastid contamination, although we cannot exclude it corresponding to a very weak mitochondrial expression, in agreement with *ccmC* processing. In Arabidopsis, processing of the *ccmC* transcript also results in a truncated orf, but in Arabidopsis the tRNACys sequence has evolved to become a pseudogene that just acts as a t-element for RNA processing [56]. Thus, it is likely that the plastid-like tRNACys of potato mitochondria is dispensable for mitochondrial translation and has been retained just as an RNA processing signal. Surprisingly, apart from tRNACys involved in *ccmC* 3’ processing, none of the other events of processing involving a tRNA sequence in potato is conserved in Arabidopsis. The reciprocal is also true, and none of the tRNA and t-elements identified as signals for processing of Arabidopsis transcripts are conserved in potato (Table 3). Thus, the evolution of transcription units and of the signals required for their processing is a very rapid process. This is in striking contrast to the very low synonymous substitution rates of the gene coding sequences [57].

It has been shown that the 3’-end of several plant mitochondrial transcripts can have stable double or single stem-loop structures, probably required for their stability or processing by RNase Z [56]. Analysis of predicted RNA structures at the transcripts 3’ ends revealed eight of such putative structural elements (Table 3). These elements are also not conserved between potato and Arabidopsis mitochondria.

### 2.4. RNA Editing

To identify editing sites, a conservative approach was used by only counting sites (C-to-T or G-to-A differences with respect to the genomic sequence, depending on the transcripts orientations) identified in at least 10% of the reads and in at least two of the three RNA-seq libraries. These were validated by visual inspection of the mapped reads. A total of 799 RNA editing sites were thus identified, a much larger number than that found in other flowering plants. That is because the very high coverage enabled the identification of many partially edited sites (Table S5). Of these, 510 (64%) were edited with an efficiency above 95%, while 149 (18%) with an efficiency below 25%. Six hundred and seventy-five (84%) of the editing sites fell into gene coding sequences (Table S5). Editing sites found in intragenic regions and in 5’- and 3’-UTRs were, in their majority, edited with low efficiency. A possible explanation is that these editing sites are off-targets poorly recognized by PPR proteins specific for other more essential editing sites in gene coding sequences.

While most editing sites result in changes in the identity of the coded amino acids, in several cases, editing is also responsible for the creation of initiation and/or termination codons. Thus, in potato, editing creates the initiation codons of *cox1* and *rps10*, although in the latter case it was shown that translation initiates at a genomic-encoded AUG and not at the conserved AUG codon created by RNA editing [58,59,60]. The stop codons of *rps10*, *atp6* and *atp9* are also created by editing, shortening the coding sequences by 10, 13 and 3 codons, respectively, as compared with the genomic orfs [58,61]. As a consequence, the N-terminal sequence of COX3 and the C-terminal sequences of ATP6 and ATP9 are the same as the corresponding Arabidopsis proteins. Surprisingly, an editing site creates a stop codon in the middle of the *ccmB*. This site was observed in 48% of the mapped reads, suggesting that about 50% of *ccmB* transcripts cannot be translated into functional proteins. A premature stop at codon 96 was also found in 26% of the reads of *sdh4*. These two cases might be additional examples of “sloppy” recognition of editing sites by PPR proteins. An interesting result concerned the *rpl16* transcript. In all higher plant mitogenomes already sequenced the *rpl16* sequence overlaps the 3’-end of *rps3* [62]. The overlapping of *rps3* and *rpl16* was also observed in the mtDNA of the liverwort *Marchantia polymorpha* [63]. It was therefore accepted that translation of *rpl16* initiated internal to the *rps3* sequence, in a different frame. However, in potato, an editing site (in 96% of the reads), that in *rps3* transforms an UCA serine codon into a UUA leucine codon, creates an internal UAG stop codon in the *rpl16* orf. This editing event was also found in Arabidopsis [64], but had not been discussed. As proposed for Marchantia, translation of *rpl16* might initiate at the level of the valine GUG codon at position 28 of the orf, which perfectly aligns with the N-term of bacterial Rpl16 [63]. Similarly, translation of *rps4* possibly initiates at the level of a GUG codon, because the initiation codon predicted in other species to be created by editing is not so, according to our experimental data. The protein would extend 84 codons upstream the first AUG and better align with *Rps4* from other species. Two editing sites found upstream of the genomic AUG are then internal to the gene sequence and required to code for conserved amino acids.

Editing also affects non-coding sequences. Thus, our data confirmed the editing of tRNAs trnF(GAA) and trnC(GCA) [65,66]. That we could detect such editing events in our libraries of 100 bp confirms that editing of tRNAs occurs at the level of the precursor transcripts. Intron sequences are also important targets of the editing machinery. There are 24 introns found in 10 potato mitochondrial genes, and as in all flowering plants these introns are members of the group II ribozyme family. As so, they share characteristic structural domains, in particular, stems V and VI at the intron 3’ border. In eight of the introns, we found editing events required for proper base-pairing of stems V and/or VI (Figure S6). Five of these were already observed and described in wheat mitochondria [67]. The relative important number of introns that require editing for proper folding suggests that timely expression of many genes is controlled by editing factors (PPR proteins) that are indirectly needed for intron splicing.

**Figure 3:**
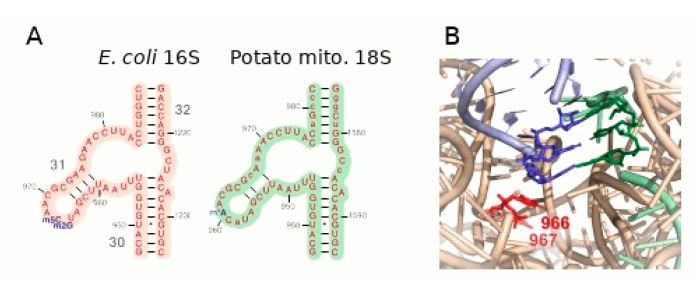
Methylation of the 18S rRNA. RNA-seq data revealed in about half of the reads an A-to-T mismatch at position 960 of 18S rRNA (position 968 in domain 31 of *E. coli* 16S rRNA). Such mismatch is diagnostic of mis-incorporation during cDNA synthesis because of m^1^A methylation. (**A**) Comparison of the corresponding domains of the *E. coli* 16S rRNA (which is methylated at positions 966 and 967, m^2^G and m^5^C respectively) and of the potato 18S rRNA. Sequence differences are in lower case. (**B**) In the 3D structure of the bacterial ribosome, these nucleotides are close to the anticodon of the tRNA at position P (dark blue nucleotides in the tRNA structure pairing with the mRNA), with base 966 stacking with the first anticodon nucleotide.

Finally, in 55% of the reads a base in the 18S rRNA sequence is a T, while an A is found in the genomic sequence (Figure S7). Such mismatch is diagnostic of a mis-incorporation during cDNA synthesis in front of a m^1^A nucleotide [68]. This methylated residue is found in the loop of a conserved stem of the rRNA. In the 16S rRNA of *E. coli*, there are two methylated nucleotides in this loop (Figure 3A), in the close vicinity of the anticodon of the tRNA at the P site, with one of the methylated bases stacking with the nucleotide at the wobble position of the anticodon (Figure 3B). The structure of the plant mitochondrial ribosome has still not been determined with sufficient resolution, but it is reasonable to speculate that methylation of this adenosine of the plant mitochondrial 18S rRNA is important for proper tRNA positioning and efficient translation.

### 2.5. Comparative Analysis of Mitochondrial Genomes among Solanaceae

The *S. tuberosum* mitochondrial genome was compared with those of other Solanaceae species available in GenBank (see Methods). Solanaceae mtDNA varies in size from 423.60 kb (*S. pennellii*) to 511.53 kb (*C. annuum*) and has similar gene content (Table 4). Following the comparison of protein-coding genes between Solanaceae species and other Angiosperms [69], we found that several genes encoding ribosomal proteins (e.g., *rpl6*, *rps2*, *rps7*, *rps8* and *rps11*) were missing in all Solanaceae species under investigation, while *rps14* is a pseudogene in all species. Similarly, *rpl6* and *rps8* are missing in rice and *A. thaliana* mitogenomes [70,71]. Likewise, the presence of a *rps14* pseudogene has been previously described in several plant mitogenomes [44,72,73,74,75,76,77], suggesting that retention of ribosomal protein genes in the mitochondrial genome is not strictly necessary for organelle function [71]. The variability in ribosomal genes composition in Angiosperms is probably due to horizontal gene transfer [78] and led to several genome outcomes [79,80]. As already observed in rapeseed and maize mitogenomes [81,82], cultivated potato has an additional copy of *cox2*, *nad1e*, *rps3*, *rps19* and *rpl16*; the duplication of these latter two genes is shared with wild and cultivated tomato species. The absence of these duplications in *S. commersonii* is probably due to an incomplete assembly and annotation of its mitogenome. As shown in Table 4, we also surveyed the transcribed orfs identified in potato with the purpose of distinguishing highly conserved and functional ones. Three out of 18 orfs were present in all analyzed Solanaceae mitogenomes, six were common to species of the *Solanum* genus, six were present in all species except *H. niger*, one was potato-specific (wild and cultivated species), and two were specific of *S. tuberosum* (*orf125* and *orf137*). In silico analyses demonstrated that three orfs, namely *orf118*, *orf306* and *orf320*, code for chimeric proteins. In particular, ORF118 includes the C-terminus of ATP6, whereas ORF306 and ORF320 carry the N-terminus of RPS1 and ATP1, respectively. Furthermore, all chimeric ORFs have predicted transmembrane helices, a typical feature of CMS-associated proteins. Transmembrane helices were also predicted in other non-chimeric ORFs (e.g., ORF125, ORF137, ORF210, ORF247, etc.) that could potentially have a role in CMS as already observed in CMS-rice [83].

**Figure 4:**
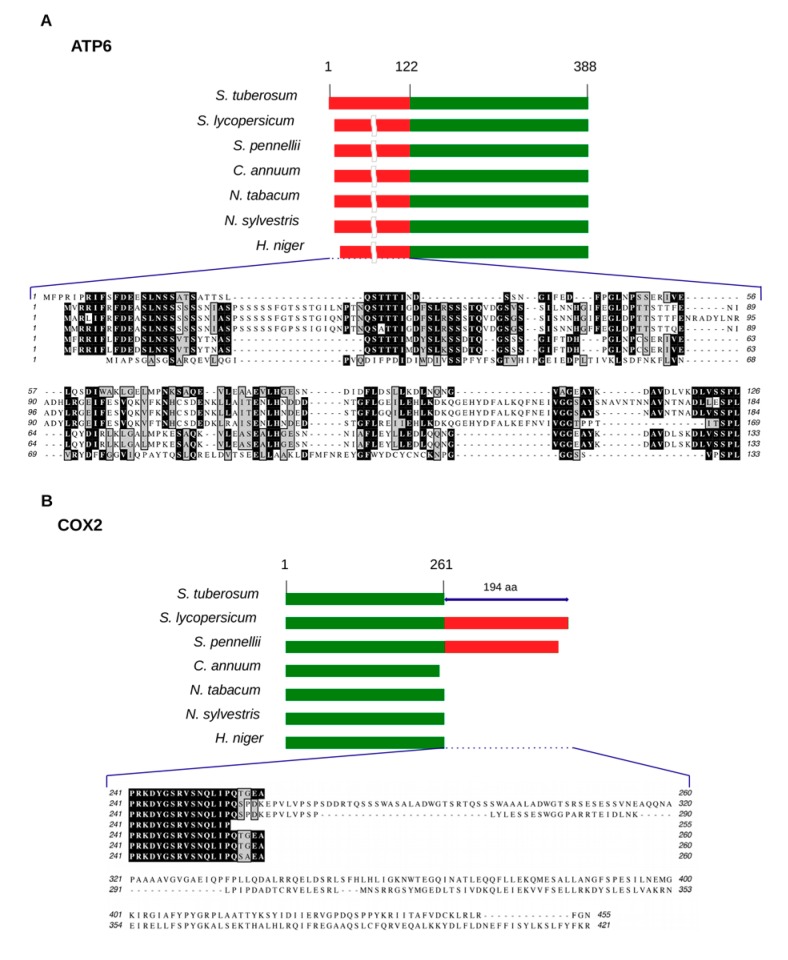
Representative examples of variable mitochondrial protein sequences in Solanaceae species. The primary structures of *S. tuberosum* ATP6 (**A**) and COX2 (**B**) proteins were compared with those of other Solanaceae species available in GenBank. (A) Red and green bars indicate non-conserved or conserved aa sequences in ATP6, respectively. (B) Cultivated (*S. lycopersicum*) and wild (*S. pennellii*) tomato have additional 194 aa at the C-terminus of the protein compared with potato and other Solanaceae species. The numbers above red and green bars indicate the start and end positions of potato mitochondrial proteins.

Potato mitochondrial proteins generally showed a highly conserved primary structure in comparison with other Solanaceae, with few exceptions (Figure 4). ATP6, a subunit of the F_0_ component of ATP synthase (respiratory complex V), differs in its N-terminal sequence, which corresponds to the leader peptide, cleaved by a metalloprotease after its translocation into the mitochondrial inner membrane [84,85,86]. This variability is because of DNA insertions and deletions, and in agreement with a faster evolution of functionally less important parts of a protein compared with those directly involved in the assembly of the ATP synthase complex [87,88]. Different N-terminal ATP6 sequences have been found in different species and even in the same species, as is the case with the two copies of ATP6 coded by the Arabidopsis mtDNA. Potato COX2, a subunit of the proton-pumping cytochrome c oxidase (respiratory complex IV), is shorter by 194 aa at the C-terminus, as compared to the predicted COX2 of *S. lycopersicum* and *S. pennellii*, and this feature is shared with *C. annuum*, *Nicotiana* species and *H. niger* (Figure 4). It is possible that in the other Solanaceae this C-terminal extension is post-translationally processed or is not translated due to the creation of a stop codon by editing. We cannot exclude, however, that variability in the primary structure of those proteins might be due to errors in the assembly process or mistakes in the annotation of the sequences.

To investigate mitochondrial genome rearrangements across cultivated potato and the other Solanaceae species, we identified syntenic blocks ≥5 kb and sequence identity ≥95%. *S. tuberosum* mtDNA shared the highest number (up to 29) of syntenic blocks with cultivated and wild tomato species (Table S6), followed by *S. commersonii*, *C. annuum* and *Nicotiana* species. Comparison with *H. niger* resulted in only two syntenic blocks. Furthermore, the syntenic blocks identified within species of the *Solanum* genus were bigger in size (between 6.7–58.3 kb) than those identified in other genera of the Solanaceae family. The largest syntenic block, about 58 kb, is shared with *S. commersonii* and *S. lycopersicum* (Figure S8). Within this syntenic block, two regions (i.e., *trnY*, *trnN*, *trnC*, *nad2* and *trnP*, *trnF*, *trnS*) were conserved with wild beet, *Cucurbita pepo* and diploid cotton *Gossypium raimondii,* and with wild beet, *G. raimondii* and coconut, respectively [3,89,90]. Two syntenic blocks containing *rps19*, *rps3*, *rpl16*, *cox2* and *trnW*, *trnP*, *nad9* and *trnH* genes are shared among species of the *Solanum* genus and *C. annuum.* Limited regions from these blocks (*rps3*, *rpl16* and *cox2* or *trnW*, *trnP*, *nad9*) were also conserved in *G. raimondii* and wild beet, respectively [89,91].

**Figure 5:**
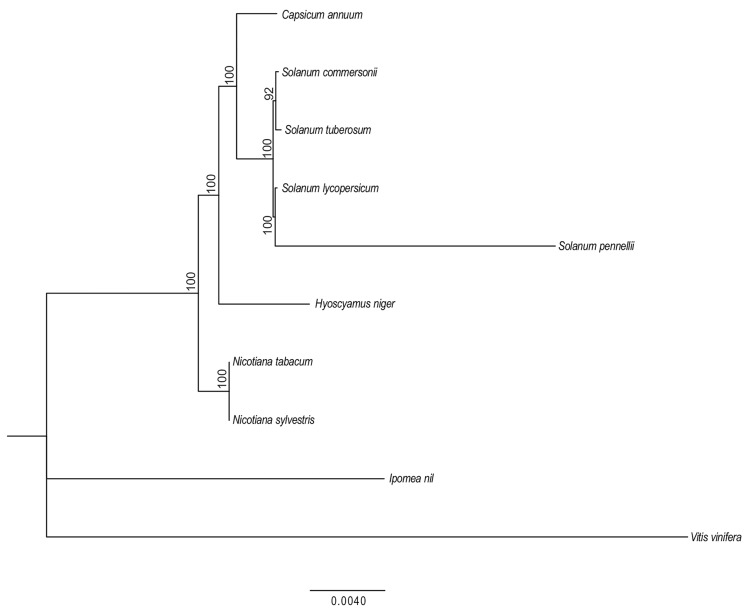
Phylogenetic tree of Solanaceae species. Phylogram of the best maximum-likelihood (ML) tree as determined using the RAxML software from the concatemer of the coding sequences of 15 protein-coding genes. Numbers associated with branches are ML bootstrap support values. *Vitis vinifera* and *Ipomea nil* were used as outgroups.

Since phylogenetic analysis of organellar genomes can identify evolutionary relationships accurately, a maximum likelihood phylogenetic tree was constructed by aligning the sequences of 15 protein-coding genes [92] from the available Solanaceae mitogenomes using *Ipomea nil* and *Vitis vinifera* as outgroup species. As expected, the sister relationships between wild and cultivated potato (*S. commersonii* and *S. tuberosum*), *S. lycopersicum* and *S. pennellii*, and between *N. tabacum* and *N. sylvestris* were all strongly supported (Figure 5). Furthermore, according to this phylogenetic reconstruction, potato species are closer to tomato species than to other Solanaceae and together form a strongly supported monophyletic lineage. The *Capsicum* genus is the closest relative to this lineage. Definitely, our results based on mtDNA sequences support the phylogenetic tree based on cpDNA sequences (i.e., combined *ndhF* and *trnL-trnF* regions) from 195 taxa [93].

## 3. Materials and Methods

### 3.1. Plant Material

The *Solanum tuberosum* subsp. *tuberosum* cv. Désirée was micropropagated in vitro, transplanted in soil and grown in greenhouse. The *Solanum tuberosum* subsp. *tuberosum* cv. Cicero was obtained from a Strasbourg (France) local farmer.

### 3.2. Isolation of mtDNA

Mitochondria were isolated from Désirée and Cicero tubers according to Pujol et al. [94] with some modifications. Briefly, after differential centrifugation mitochondria was purified on discontinuous Percoll gradient (14-28–45% *v*/*v*) and centrifuged at 70,000× *g* for 45 min (Beckman SW28 rotor). Mitochondria were collected at the 28–45% Percoll interface and washed twice with five volumes of washing buffer without BSA and centrifuged for 15 min at 10,000× *g*. Mitochondria were treated with DNase I for 45–60 min at 37 °C (1 mg/200 g of potato tubers, in 0.3 M sucrose, 50 mM Tris-HCl pH 7.5, 10 mM MgCl_2_), after which the reaction was diluted with three volumes of washing buffer without BSA and centrifuged for 20 min at 12,000× *g*. The mitochondrial pellet was washed twice with five volumes of washing buffer without BSA and the mtDNA extracted as previously described [95].

### 3.3. Genome Sequencing and Assembly

Désirée. Sequencing was performed using both Illumina (Illumina, San Diego, CA, USA) and PacBio RS II single-molecule real-time (SMRT) sequencing technology (Pacific Biosciences, Menlo Park, CA, USA). A single sonication step was used for shear input DNA used for library construction with the TruSeq DNA kit (Illumina, San Diego, CA, USA). The sequencing (2 × 250 bp) was performed on a MiSeq device (estimated insert size ~500 bp).

FastQC (http://www.bioinformatics.babraham.ac.uk/projects/fastqc/), FASTX-Toolkit (http://hannonlab.cshl.edu/fastx_toolkit/) and Trimmomatic [96] were combined for assessing the overall quality of the sequencing run, to trim off poor quality bases, and to filter high-quality scores. High-quality reads were de novo assembled with Velvet (k-mer size 73) [97], filtering out contigs <200 bp in length. PacBio and Illumina data were used together to generate the final assembly. The hybrid scaffolding strategy was used. Briefly, using the existing assembly based on Illumina reads, PacBio sequences were used to join contigs with the AHA scaffolding algorithm [98] that is available through the SMRT analysis package (version 2.0, Pacific Biosciences). Assembled scaffolds were gap-filled and further scaffolded using GapCloser [99] with Illumina reads.

Cicero. Sequencing was performed using PacBio RS II single-molecule real-time (SMRT) sequencing technology (Pacific Biosciences, CA). PacBio data was assembled using the SMRT analysis package (version 2.2, Pacific Biosciences). The minimum coverage for correction was set to 35, and the minimum seed read length was set to 7 kb. The approximate genome size for the assembler module was set to 500 kb. Canu version 1.4 [100] was also used in order to inspect overlaps between corrected reads (extracted from the SMRT pipeline). Bandage [101] was used to draw overlapping read graphs. Contigs corresponding to plastid sequences were removed. Depth of sequencing coverage (Figure S9) was evaluated by mapping raw reads (prior to the correction step) to the assembled mitogenome. Possible configurations involving circularization of contigs or insertion of one into another by recombination were confirmed by PCR across the contig borders or across the repeated sequences involved in recombination, respectively.

### 3.4. Genome Annotation

Auto-annotation was performed to identify most known genes by comparison with the tobacco mtDNA annotations (GenBank: NC_006581.1). Annotations were then manually curated using the MacVector (MacVector, Inc., version 16.0.10) annotation tool.

### 3.5. Transcriptome Sequencing and Analysis

Total RNA extracted from pure mitochondria of the Cicero accession was sequenced as described elsewhere [102]. Briefly, RNAs were extracted with Tri Reagent^®^ (Molecular Research Center, Cincinnati, OH, USA) according to the manufacturer’s instructions and were DNase-treated with DNase RQ1 (Promega, Madison, WI, USA). The RNA-seq libraries, corresponding to three biological replicates, were prepared by the IGBMC Microarray and Sequencing Platform (Strasbourg, France) following Illumina protocols. The libraries were sequenced on an Illumina Genome Analyser IIx as paired-end 100 bp reads. A quality check on the raw data was performed using FastQC. Reads were aligned to the mtDNA sequence using Hisat2 version 2.0.5 [103]. Very high coverage was obtained: 61 out of 94, 29 out of 86 and 39 out of 76 million reads could be aligned to the mtDNA sequence.

### 3.6. Detection of Repeats

Repeats were searched by comparing the genome against itself using the Pustell DNA Matrix (Dot Plot) function of the MacVector package, parameters set to window size 30 bp, minimum score 90%, Hash value 8. Repeats of length greater than 100 bp were kept (Table S2). Primer3 included in the MacVector package was used for the design of PCR primers (Table S3).

### 3.7. Comparative and Evolutionary Analysis

The *S. tuberosum* mitogenome was compared with seven already published Solanaceae mitogenomes: *Solanum commersonii* (GenBank: MF989960.1, MF989961.1), *S. lycopersicum* (GenBank: NC_035963.1), *S. pennellii* (GenBank: NC_035964.1), *Nicotiana tabacum* (GenBank: NC_006581.1), *N. sylvestris* (GenBank: NC_029805.1), *Capsicum annuum* (GenBank: NC_024624.1), *Hyoscyamus niger* (GenBank: NC_026515.1). Syntenic blocks between mitogenomes where identified by BLASTn searches (e-value ≤ 1 × 10^−5^) based on the size of the alignment ≥5 kb and on sequence identity ≥95%. A maximum likelihood phylogenetic tree was constructed using the seven Solanaceae species aforementioned and two species, *Ipomea nil* (GenBank: NC_031158) and *Vitis vinifera* (GenBank: NC_012119), as outgroups. For comparison the coding sequences of 15 conserved protein-coding genes (*atp1*, *atp9*, *ccmB*, *cob*, *cox1*, *cox3*, *nad1*, *nad3*, *nad4*, *nad4L*, *nad6*, *nad7*, *nad9*, *rps3*, *rps4*) were extracted and a concatemer was assembled for each species. Sequences were then aligned in MAFFT v.7 [104] using default settings. RAxML v.8.2.12 [105] with the ‘GTRGAMMA’ evolutionary model under the rapid bootstrap algorithm with 1000 replicates was used to represent evolutionary relationships among species.

### 3.8. Data Deposition

The two mitogenomes reported in this paper were deposited in GenBank under accession numbers MN114537, MN114538, MN114539 (Cicero) and MN104801, MN104802, MN104803 (Désirée). Désirée raw sequences are available at the Sequence Read Archive (SRA) under the BioProject ID PRJNA544585. Raw RNA-seq data can be found in the Gene Expression Omnibus archive under the accession number GSE91388.

## 4. Conclusions

In the present study, we were able to resolve the mitogenome assembly of potato in 3 structural units by using long and short read sequencing data. The potato example adds to the few multichromosomal plant mitogenomes that cannot be resolved into a single circular map [2,11,12,14,15,16]. Isoforms due to recombination could be detected and confirmed by PCR. Long read sequences were thus very informative for better apprehending the recombination dynamics of plant mitogenomes. Comparative and phylogenetic analyses allowed us to identify large syntenic blocks among Solanaceae species.

We also provided a full picture of RNA processing using RNA-seq data, revealed numerous partially edited sites both in exon and intron sequences, potentially important methylation of the 18S rRNA and active transcription of several orfs of unknown function.

The detailed information obtained in this study for common potato will be very useful in future comparative analyses with mitogenomes of other tuber bearing species in order to better comprehend the co-evolution of nuclear and cytoplasmic genomes in this group of species. In particular, precise comparison of mitochondrial orfs, differentially present and/or expressed in various potato species as well as in rearranged mitogenomes of somatic hybrids, is of paramount importance to investigate molecular bases of nuclear-cytoplasmic interactions leading to cytoplasmic male sterility in some *Solanum* tuber-bearing interspecific hybrids [39]. The identification of mtDNA sequences involved in such a trait will be critical for developing a novel system to induce CMS also in other crops.

## Figures and Tables

**Table 1:** List of transcribed protein-encoding genes and orfs. Genes are clustered according to transcription units.

**Molecule 1**				
**Gene**	**Start**	**Stop**	**Strand**	**Comments**
nad1e	3860	4118	+	
orf119	12570	12929	–	
atp1	13197	14732	–	
Mttb	31292	32122	–	
orf265a	32244	33041	–	N-term atp8 (20 codons), ELF-domain (pfam03317). Co-transcribed with MttB
26S	33385	36879	–	
nad2cde	50394	50581	–	
	52050	52622	–	
	55094	55254	–	
nad5ab	61695	62910	–	
	63755	63984	–	
nad4	64989	65077	–	
	67705	68127	–	
	71259	71773	–	
	73185	73645	–	
orf125	73849	74226	–	
orf247	78169	78912	–	
rps4	90192	91013	+	No evidence of TAG created by editing. Possible non-canonical initiation at GTG codon
nad6	91707	92360	+	Transcript is processed upstream of stop codon
nad4L	98027	98329	+	
atp4	98518	99114	+	
orf438	109344	110660	–	RdRp-like
orf141	110784	111209	–	
5S	111720	111838	–	
18S	112001	113946	–	
orf304	117275	118189	+	N-term pfam12725. C-term has 59% identity to hypothetical protein RirG_027070
nad1d	118391	118449	+	
matR	119111	121087	+	
nad5de	125647	125793	–	
	126889	127283	–	
orf152	127397	127855	–	
orf105	128459	128776	–	
orf159	128839	129318	–	RdRp-like
orf137	139197	139610	–	CMS-associated protein
nad1a	148955	149339	+	
rps19	159585	159869	+	
rps3	159883	159956	+	
	161026	162643	+	
rpl16	162534	163049	+	Editing site 162570 (96%) creates internal stop codon. Editing is conserved in Arabidopsis. It implies that there is no re-initiation of translation inside rps3. Possible initiation at GTG codon.
cox2	163299	163680	+	
	165066	165466	+	
ccmC	172753	173583	+	ORF overlaps with tRNA and transcript is processd at the tRNA 5’, without stop codon.
rps19	208754	209038	+	
rps3	209052	209125	+	
	210195	211812	+	
rpl16	211703	212218	+	
cox2	212468	212849	+	
	214235	214635	+	
orf77	233142	233375	+	
nad1e	233431	233689	+	
atp6	233940	235106	+	Editing site 235065 creates stop codon making orf 13 aa shorter, with the same C-term as Arabidopsis atp6.
atp9	243146	243379	+	Editing site 243368 creates stop codon making protein 3 aa shorter. With editing it is the same C-term as Arabidopsis atp9.
nad5c	246225	246246	+	
orf161	246314	246799	+	
nad7	271765	272026	–	
	273770	274477	–	
	275936	276004	–	
	276920	277062	–	
orf103	278419	278730	–	
nad1bc	292250	292441	–	
	293925	294007	–	
rps13	294547	294897	–	
nad1d	295623	295681	–	
orf304	295883	296797	–	hypothetical protein RirG (*Rhizophagus irregularis*)
18S	300126	302071	+	
5S	302234	302352	+	
orf141	302863	303288	+	Similarities to region 3’ UTR of orf247
orf438	303412	304728	+	RdRp-like
orf320	310786	311748	–	Chimeric orf: 5’ of atp1, 3’ region upstream nad5c. Promoter of atp1 that is present in repeat R5
**Molecule 2**				
**Gene**	**Start**	**Stop**	**Strand**	**Comments**
ccmFC	6136	6685	–	
	7635	8401	–	
Cob	33524	34705	–	
sdh4	51176	51589	–	Overlaps cox3. Real ATG might be at codon 24. The transcript is processed about 8 codons before stop codon. Internal stop codon created by partial editing (26%) at codon 93.
cox3	51517	52314	–	
atp8	52947	53417	–	
orf118	54391	54747	–	Chimeric orf: C-term is from atp6
rps1	54964	55635	–	
ccmFN	71926	73737	–	
cox1	76329	77825	–	Initiation codon created by editing
rps10	78075	78187	–	Stop codon created by editing (78107)
	78963	79212	–	Initiation codon created by editing (79211).
rps14 *	81682	82051	–	Pseudo-gene
rpl5	82053	82613	–	
rps12	99496	99867	–	
nad3	99916	100272	–	
orf265b	100423	101220	–	N-term atp8, ELF-domain (pfam03317)
**Molecule 3**				
**Gene**	**Start**	**Stop**	**Strand**	**Comments**
ccmB	9945	10565	+	Editing site 10238 creates stop codon in about 50% of the transcripts, in the middle of the ORF.
rpl10	16440	16919	–	
rpl2	17204	17320	–	
	19230	20114	–	
orf210	20294	20926	–	
sdh3	35400	35726	+	
nad2ab	36302	36453	+	
	37470	37862	+	
nad9	41589	42161	–	

**Table 2:** List of tRNA genes. Plastid-like tRNA genes are tagged by ‘*’. Expression is according to [46]. Absence of expression for plastid-like Cys, and Val tRNA genes has been checked by northern blot (Figure S3). nd = not determined.

tRNA Gene	Start	Stop	Strand	Editing NGS	Expression
**Molecule 1**					
trnP(UGG)	17220	17294	-		+
trnF(GAA)	17545	17618	-	+	+
trnS(GCU)	17981	18068	-		+
trnMf(CAU)	37458	37531	-		+
trnY(GUA)	55772	55854	-		+
trnN(GUU) *	56390	56461	-		+
trnC(GCA)	58606	58676	-	+	+
trnC(GCA) *	171005	171076	+		-
trnI(CAU) *	173488	173561	+		nd
trnMe(CAU) *	189363	189435	-		+
trnG(GCC)	201326	201397	+		+
trnQ(UUG)	204702	204773	+		+
trnI(CAU)	260636	260709	+		+
**Molecule 2**					
trnN(GUU) *	28511	28582	+		+
trnS(UGA)	35798	35884	-		+
trnD(GUC) *	43141	43214	+		+
trnS(GGA) *	43901	43987	+		+
trnV(GAC) *	64733	64804	-		-
**Molecule 3**					
trnK(UUU)	7326	7398	-		+
trnE(UUC)	23306	23377	-		+
trnW(CCA) *	40411	40484	-		+
trnP(UGG)	40642	40715	-		+
trnH(GUG) *	45897	45971	-		+

**Table 3:** List of stem-loops and T-elements.

***Arabidopsis thaliana***				
**Gene**	**mRNA End**	**Kind of Secondary Structure**	**Putative Nuclease**	**Conservation in Potato**
ccmFC	5’	trnG	RNAseZ	No
rps3	5’	trnK	RNAseZ	No
rps4	5’	t-element	RNAseZ	No
ccmFN1	5’	t-element	RNAseZ	No
cox1	5’	t-element	RNAseZ	No
rpl5	5’	Acceptor stem-like stem–loop	RNAseZ	No
rpl5	5’	Acceptor stem-like stem–loop	RNAseZ	No
atp6-2	5’	Acceptor stem-like stem–loop	RNAseZ	No
nad7	5’	Acceptor stem-like stem–loop	RNAseP	No
atp6-1	3’	trnS	RNAseP	No
atp6-2	3’	trnS	RNAseP	No
atp9	3’	Double stem-loop	RNAseZ	No
nad1e	3’	Double stem-loop	RNAseZ	No
cox2	3’	Stem-loop	RNAseZ	No
ccmC	3’	t-element	RNAseP	trnI
nad6	3’	t-element	RNAseP	Yes
***Solanum tuberosum***				
rrrn26S	5’ precursor	trnfM	RNAseZ	
nad2cde	5’	trnY	RNAseZ	
non-coding RNA	5’	trnC	RNAseZ	
Cob	5’	trnS	RNAseZ	
ccmC	5’	trnC	RNAseZ	
ccmC	3’	trnI	RNaseP	
atp1	3’	Double stem-loop	RNAseZ	12397–12441
mttB	3’	Stem-loop	RNAseZ	31111–31148
nad5ab	3’	Double stem-loop	RNAseZ	60956–60986
orf247	3’	Double stem-loop	RNAseZ	76895–76938
nad6	3’	t-element	RNAseZ	92309–92353
atp4	3’	Stem-loop	RNAseZ	99142–99166
nad1a	3’	Stem-loop	RNAseZ	150600–150637
orf438	3’	Stem-loop	RNAseZ	305247–305291

**Table 4:** Mitochondrial genes encoding proteins and transcribed open reading frames (*orfs*) among Solanaceae species available in GenBank. The symbol ● indicates the presence of the gene; ψ, a pseudogene; -, gene loss.

Gene	*S. tuberosum*	*S. commersonii*	*S. lycopersicum*	*S. pennellii*	*C. annuum*	*N. tabacum*	*N. sylvestris*	*H. niger*
*atp1*	●	●	●	●	●	●	●	●
*atp4*	●	●	●	●	●	●	●	●
*atp6*	●	● ^a^	●	●	●	●	●	●
*atp8*	●	●	●	●	●	●	●	●
*atp9*	●	●	●	●	●	●	●	●
*ccmB*	●	●	●	●	●	●	●	●
*ccmC*	●	●	●	●	●	●	●	●
*ccmFc*	●	●	●	●	●	●	●	●
*ccmFN*	●	●	●	●	●	●	●	●
*cob*	●	●	●	●	●	●	●	●
*cox1*	●	●	●	●	●	●	●	●
*cox2*	●	●	●	●	●	●	●	●
*cox3*	●	●	●	●	●	●	●	●
*matR*	●	●	●	●	●	●	●	●
*mttB*	●	●	●	●	●	●	●	●
*nad1*	●	●	●	●	●	●	●	●
*nad2*	●	●	●	●	●	●	●	●
*nad3*	●	●	●	●	●	●	●	●
*nad4*	●	●	●	●	●	●	●	●
*nad4L*	●	●	●	●	●	●	●	●
*nad5*	●	●	●	●	●	●	●	●
*nad6*	●	●	●	●	●	●	●	●
*nad7*	●	●	●	●	●	●	●	●
*nad9*	●	●	●	●	●	●	●	●
*rpl2*	●	●	●	●	●	●	●	●
*rpl5*	●	●	●	●	●	●	●	●
*rpl10*	●	●	●	●	●	●	●	●
*rpl16*	●	●	●	●	●	●	●	●
*rps1*	●	●	●	●	●	●	●	●
*rps3*	●	●	●	●	●	●	●	●
*rps4*	●	●	●	●	●	●	●	●
*rps10*	●	●	●	●	●	●	●	●
*rps12*	●	●	●	●	●	●	●	●
*rps13*	●	●	●	●	●	●	●	●
*rps14*	ψ	ψ	ψ	ψ	ψ	ψ	ψ	ψ
*rps19*	●	●	●	●	●	●	●	●
*sdh3*	●	●	●	●	●	●	●	●
*sdh4*	●	●	●	●	●	●	●	●
*orf77*	●	●	●	●	●	●	●	-
*orf103*	●	●	●	●	●	●	●	●
*orf105*	●	●	●	●	●	●	●	-
*orf118*	●	●	ψ	ψ	-	-	-	-
*orf119*	●	●	●	●	●	●	●	●
*orf125*	●	-	-	-	-	-	-	-
*orf137*	●	-	-	-	-	-	-	-
*orf141*	●	●	●	●	-	-	-	-
*orf152*	●	●	●	●	●	●	●	-
*orf159*	●	●	●	●	ψ	-	-	-
*orf161*	●	●	●	●	●	●	●	ψ
*orf210*	●	●	●	●	-	-	-	-
*orf247*	●	●	●	●	-	-	-	-
*orf265a*	●	●	●	●	●	●	●	ψ
*orf265b*	●	●	●	●	●	●	●	ψ
*orf304*	●	●	●	●	ψ	-	-	-
*orf320*	●	●	●	●	●	●	●	●
*orf438*	●	●	●	●	-	-	-	-

^a^ = atp6 sequences available in GenBank (MF989960.1 and MF989961.1 accessions) were incomplete.

## Supplementary Materials

Supplementary materials can be found at https://www.mdpi.com/1422-0067/20/19/4788/s1.
