# Supplementary material for: Complete Sequence, Multichromosomal Architecture and Transcriptome Analysis of the Solanum tuberosum Mitochondrial Genome
Source: Int J Mol Sci. 2019 Sep 26;20(19):4788. doi: 10.3390/ijms20194788 (PMC6801519; doi:10.3390/ijms20194788)
Supplement: Supplementary file 1 [file ijms-20-04788-s001.pdf]

Supplementary material for

Complete Sequence, Multichromosomal Architecture and Transcriptome Analysis of the *Solanum tuberosum* Mitochondrial Genome

Jean-Stéphane Varré [1,†], Nunzio D'Agostino [2,†,‡], Pascal Touzet [3], Sophie Gallina [3], Rachele Tamburino [4], Concita Cantarella [2], Elodie Ubrig [5], Teodoro Cardi [2], Laurence Drouard [5], José Manuel Gualberto [5,*], Nunzia Scotti [4,*]

1 Univ. Lille, CNRS, Centrale Lille, UMR 9189—CRIStAL—Centre de Recherche en Informatique Signal et Automatique de Lille, F-59000 Lille, France
2 CREA Research Centre for Vegetable and Ornamental Crops 84098 Pontecagnano Faiano, SA, Italy
3 Univ. Lille, CNRS, UMR 8198—Evo-Eco-Paleo, F-59000 Lille, France
4 CNR-IBBR, National Research Council of Italy, Institute of Biosciences and BioResources, 80055 Portici, NA, Italy
5 Institut de Biologie Moléculaire des Plantes-CNRS, Université de Strasbourg, Strasbourg, France

* Correspondence: jose.gualberto@ibmp-cnrs.unistra.fr; nscotti@unina.it
† These authors contributed equally to this work.
‡ Present address: Department of Agricultural Sciences, University of Naples Federico II, 80055 Portici, NA, Italy

Supplementary Figure S1. Bandage graph of corrected reads from unitig 0. We took both occurrences of R1′ (corresponding to the red repeat Figure 1A) and both occurrences of R3 with 3 kbp surrounding sequences. We then mapped those 4 sequences against the Canu assembly graph. Red and black colored reads correspond to R1′ occurrences and green and blue colored reads correspond to R3 occurrences. This shows that the assembly tool has to choose between several paths in the assembly graph leading to different molecule arrangements. The orange path shows how unitig 0 has been built.

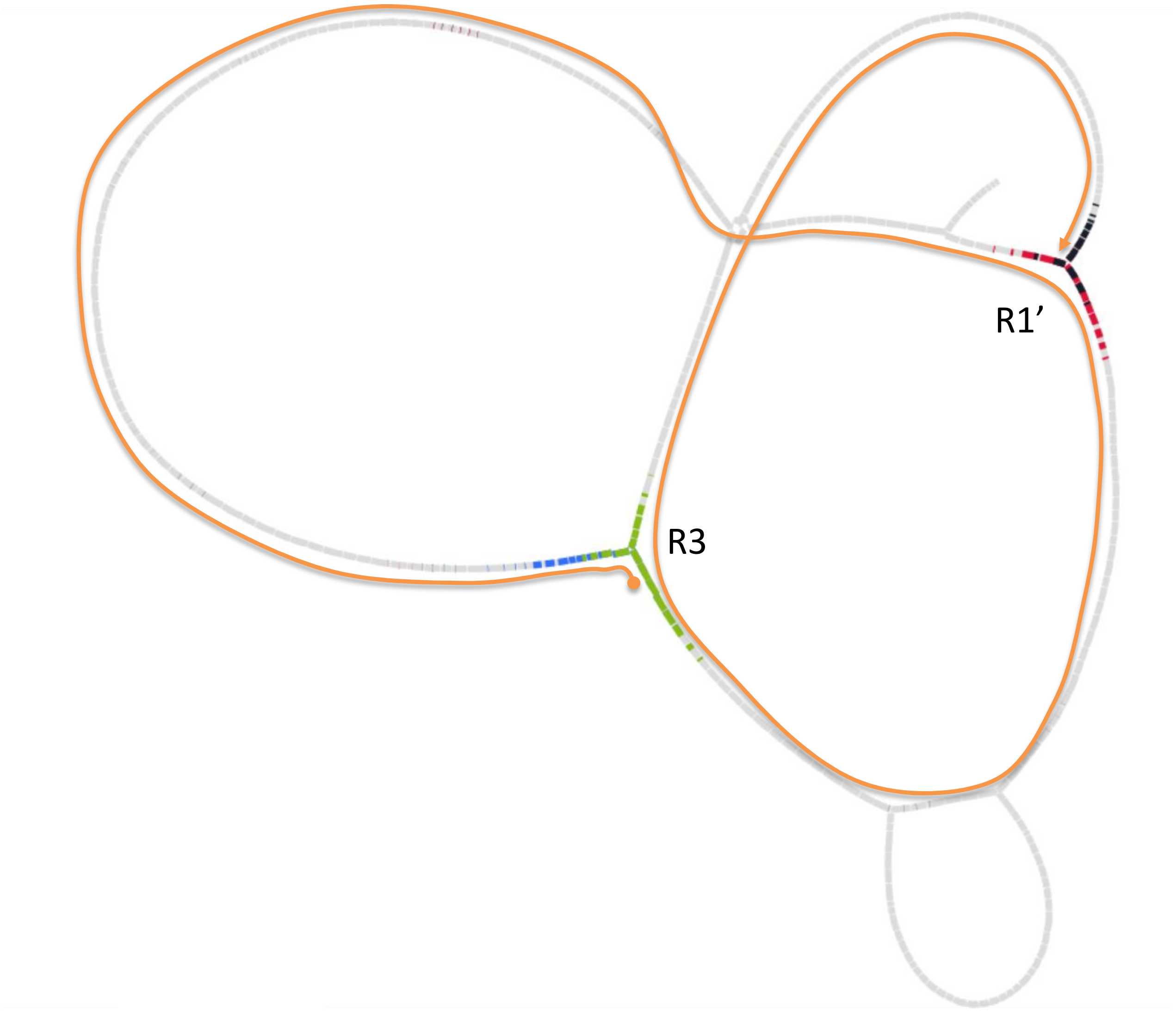

Supplementary Figure S2. A) Confirmation of assembly configurations by PCR. Primer names above the gels are those given in Supplementary Table S2 and indicated in Figure 1. M is the lane of size markers. **B)** Schematic representation of the confirmation that unitigs that display repeated sequences at their ends (black boxes) are circular, as for experiment 2. **C)** Identification of the predominant isoforms mediated by the recombination involving a repeated sequence, as for experiments 8-11. The experiments were the following, as also represented in Figure 1.

1. Confirm circularization of unitig_4
2. Confirm circularization of unitig_5 (molecule 3)
3. Confirm circularization of unitig_6

4-7. Test possible alternative configurations around repeat R1. Experiments 4 and 6 gave products of the expected sizes.

8-11. Test possible isomeric configurations around repeat R3. The very efficient amplification in lanes 9 and 11 suggest that they are the ones that exist as part of the main mitogenome.

12-15. Test that unitig_1 can exist inside unitig_0 by recombination involving repeated sequence R2.

16-19. Confirm the assembly of the sequence between repeats R1B and R5B.

20-21. Confirm the existence of the corresponding isoforms around repeats R4A and R4B, and that unitig_4 and unitig_6 can be assembled into a single circle (molecule 2) by recombination involving repeated sequences R4.

22-24. Additional tests to confirm that unitig_1 can exist inside unitig_0 by recombination involving repeated sequence R2.

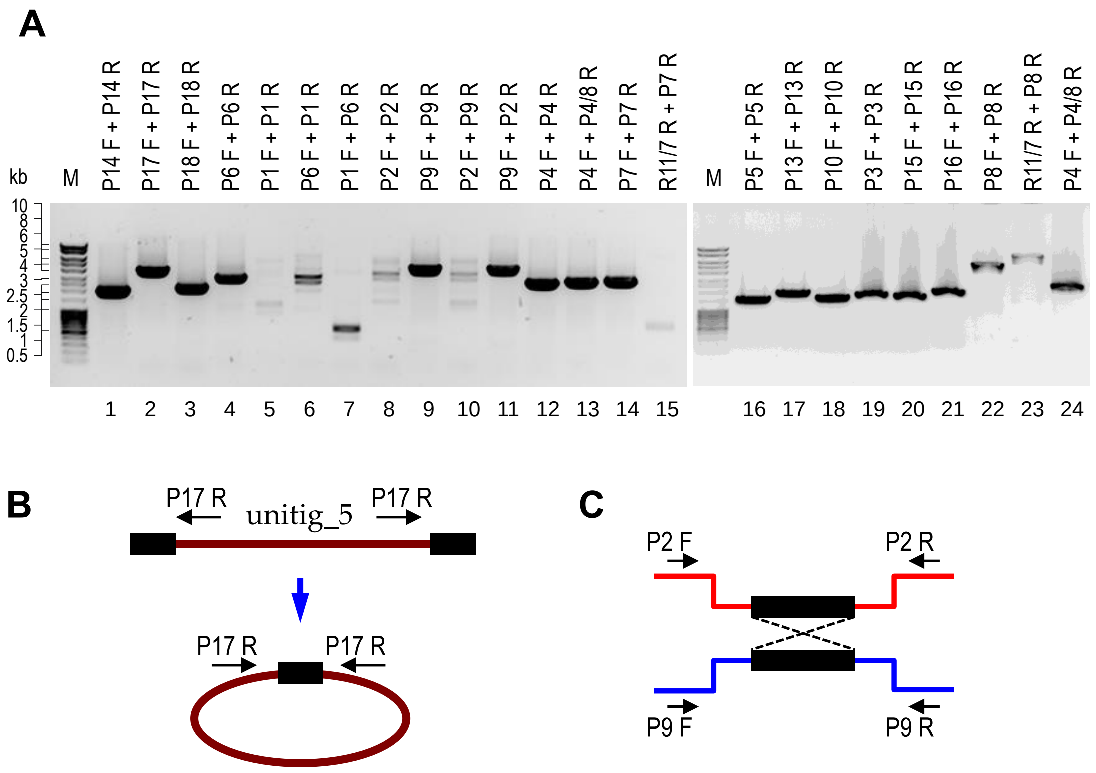

Supplementary Figure S3. Alternative scaffolding of unitigs 0, 1 and 3 leading to circular molecules.
(**A**) The first one uses repeat R3 (blue repeat) leading to a circular molecule of 229,571 bp, by removing the end of unitig 0 (see Figure 1A). (**B**) The second alternative configuration uses repeat R5 (purple repeat) leading to a slightly different circular molecule of 296,789 bp, by removing the beginning of unitig 0 (see Figure 1A).

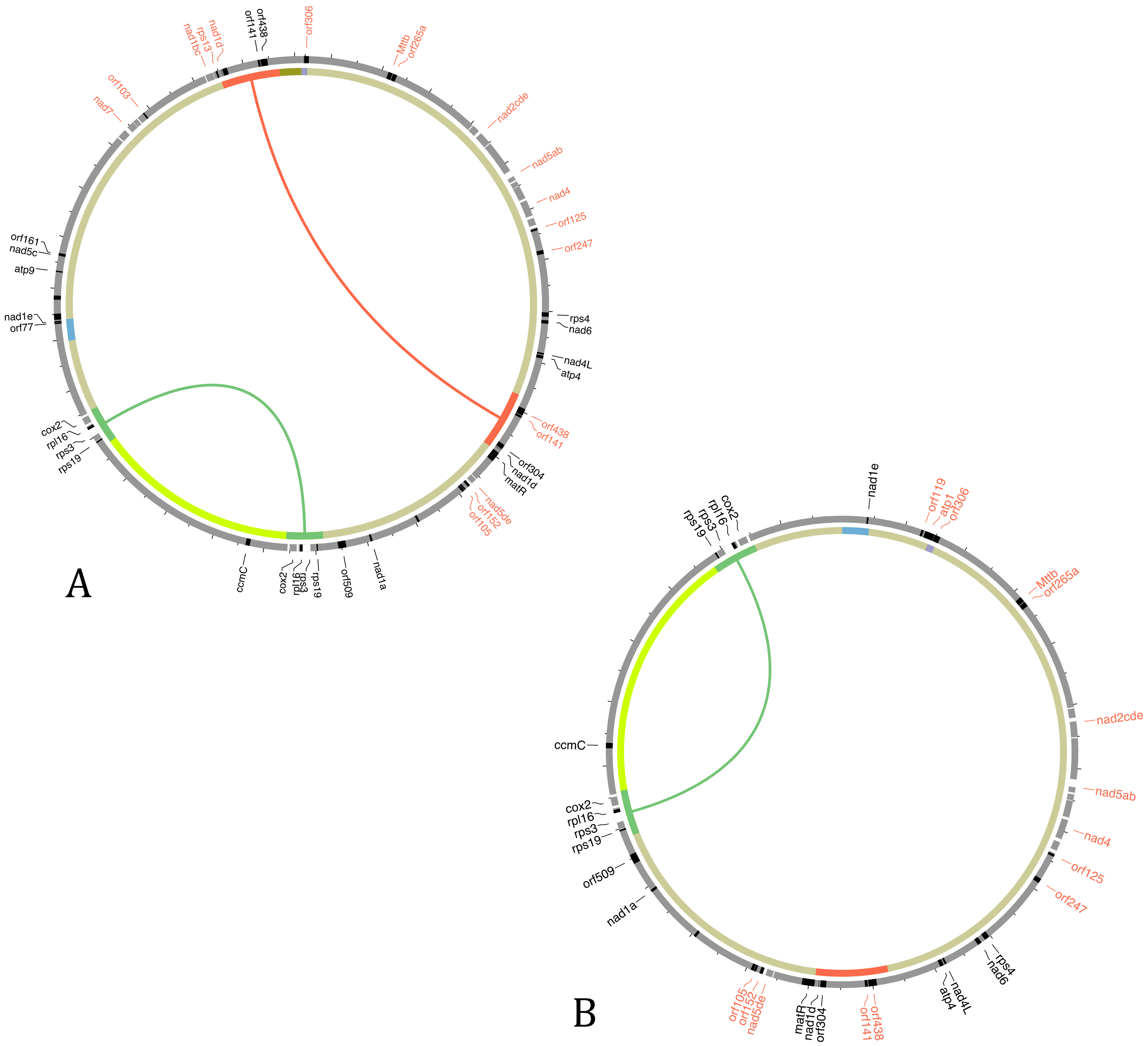

Supplementary Figure S4. Northern blots with *S. tuberosum* plastidial (P) and mitochondrial (M) tRNAs. The oligonucleotide probes are specific for plastidial tRNAHis, tRNACys and tRNAVal. His: 5′ TGGGGCGAACGACGGGAATTG 3′; Cys: 5′ TGGAGGCGACACCCGGATTTG 3′; Val: 5′ TGGTAGGGATAATCAGGCTC 3′.

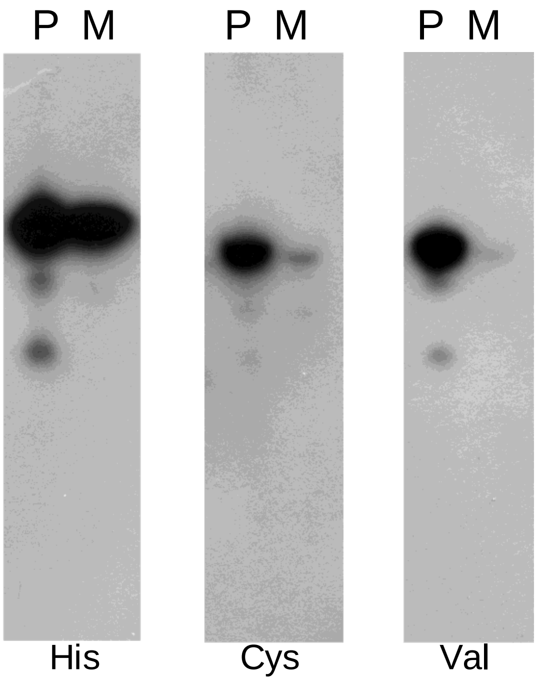

Supplementary Figure S5. (**A**) Evidence for the expression of *orf137*, previously described as potentially involved in CMS in chili pepper [52]. (**B**) Evidence for co-transcription of *orf265* with *nad3* and *rps12*.

A

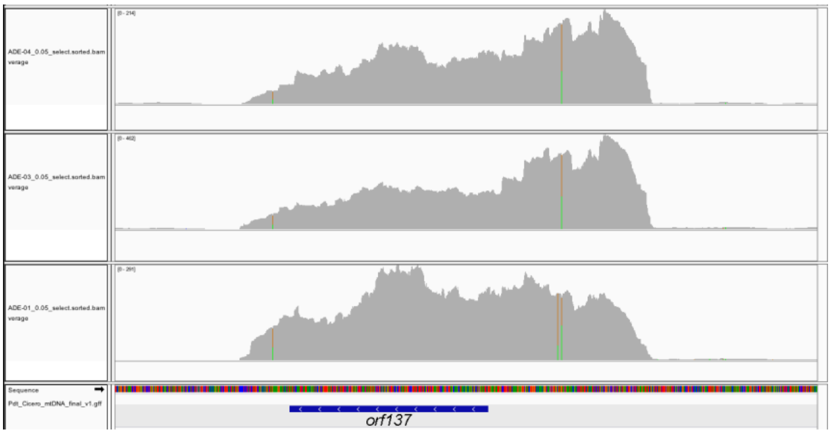

B

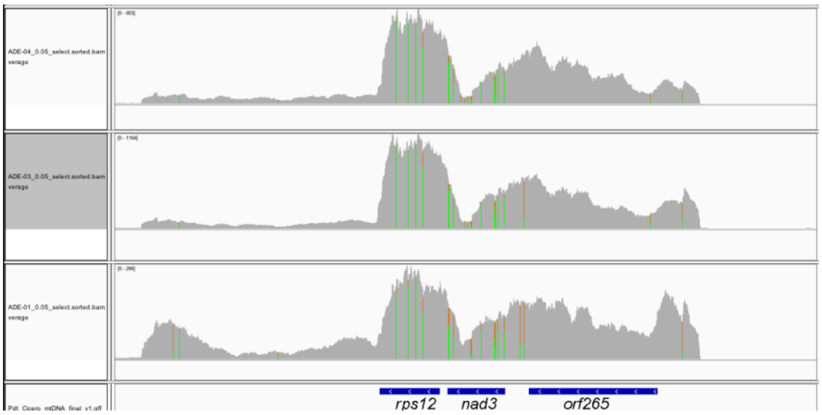

Supplementary Figure S6. Editing of intron sequences. The table shows the comparison of editing sites observed in wheat (see Ngu et al., [67]) and in potato mitochondria. The positions of sites (circled in red) only identified in potato are indicated in the predicted structures of the domains V and VI at the 3′ of the introns. The positions of the other editing sites are as described in [67]. The asterisk indicates intron 3 of potato *nad7* that corresponds to intron 4 of wheat *nad7*. The nucleotide circled in pink is edited in wheat but not in potato.

Gene Intron - Domain	Wheat	Potato
nad1 i1 (trans)- Stem VI	+	-
nad1 i3 (trans)- Stem VI	+	+
nad1 i4 (trans)- Stem VI	+	+
nad5 i1 - Stem VI	-	+
nad5 i2 (trans)- Stem VI	+	+
nad4 i3 - Stem VI	+	+
nad7 i2 - Stem VI	-	+
nad7 i3* - Stem V (a)	+	-
nad7 i3* - Stem V (b)	-	+
ccmFc i1 - Stem V	+	+

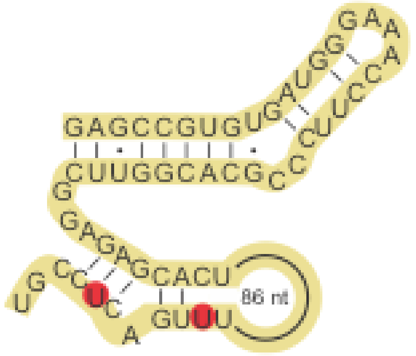

nad5 intron 1

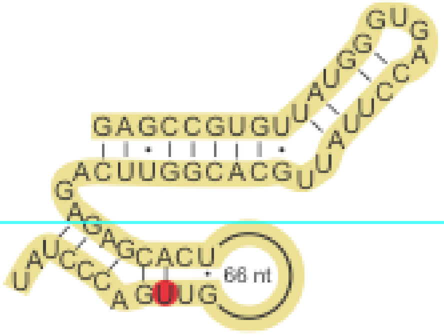

nad7 intron 2

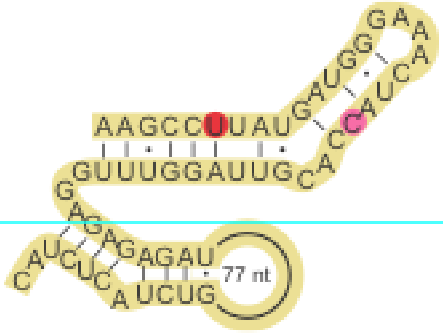

nad7 intron 3*

Supplementary Figure S7. Evidence for methylation of base A960 of rRNA 18S. An A-to-T mismatch was found in the reads of the three RNA-seq libraries.

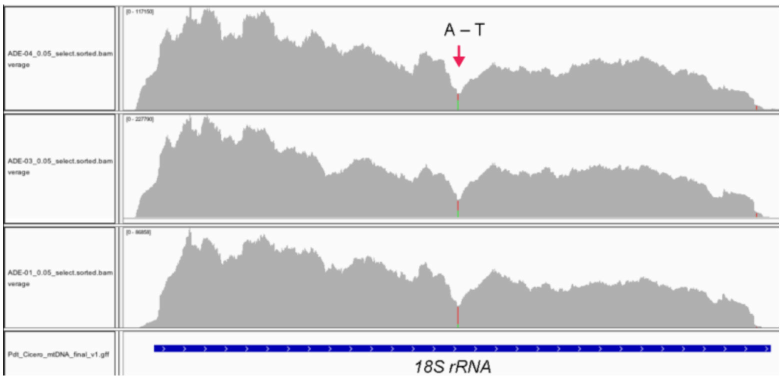

Supplementary Figure S8. Schematic representation of the largest syntenic block shared with most Solanaceae species under investigation. The red, green, yellow and light blue short lines represent the small mitochondrial sequences containing micro-homologies that could be involved in the origin of the different syntenic blocks. Dotted line represents a deletion in *S. commersonii* that interrupts colinearity with *S. tuberosum*.

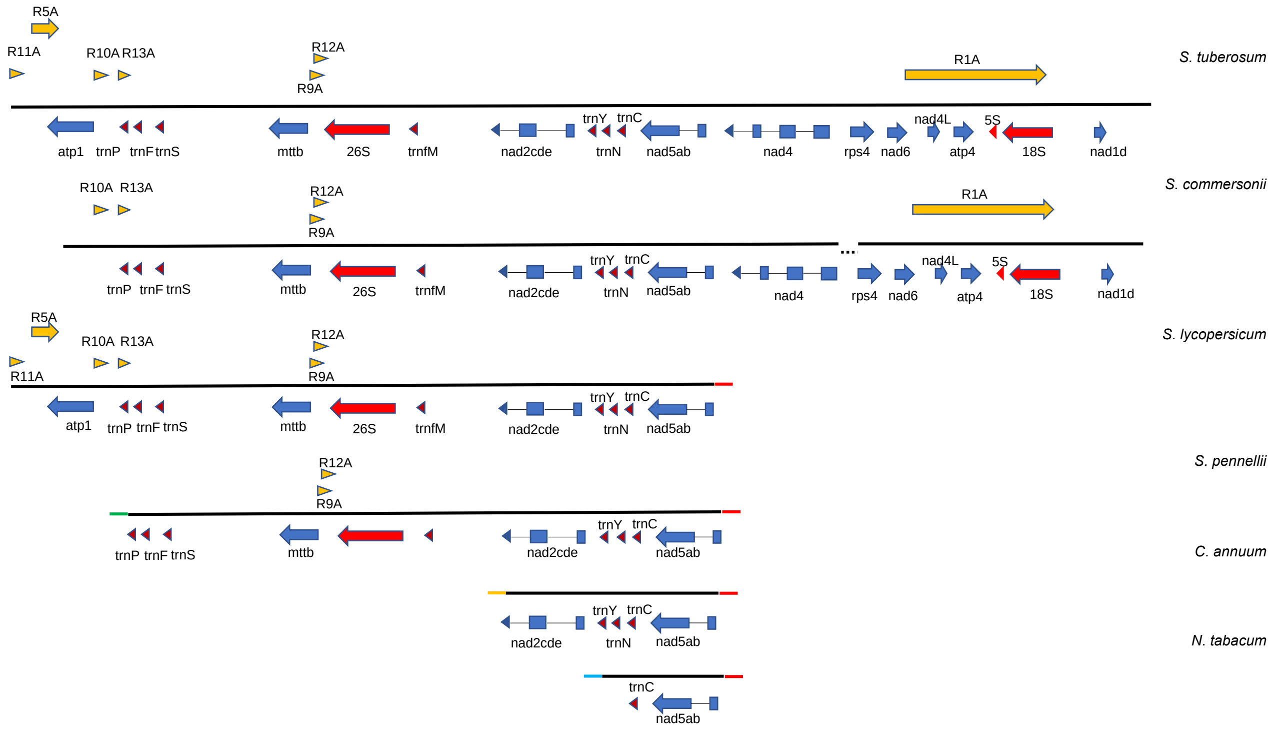

Supplementary Figure S9. Depth of coverage of PacBio raw reads. Coordinates are given relatively to the concatenation of molecules 1, 2 and 3.

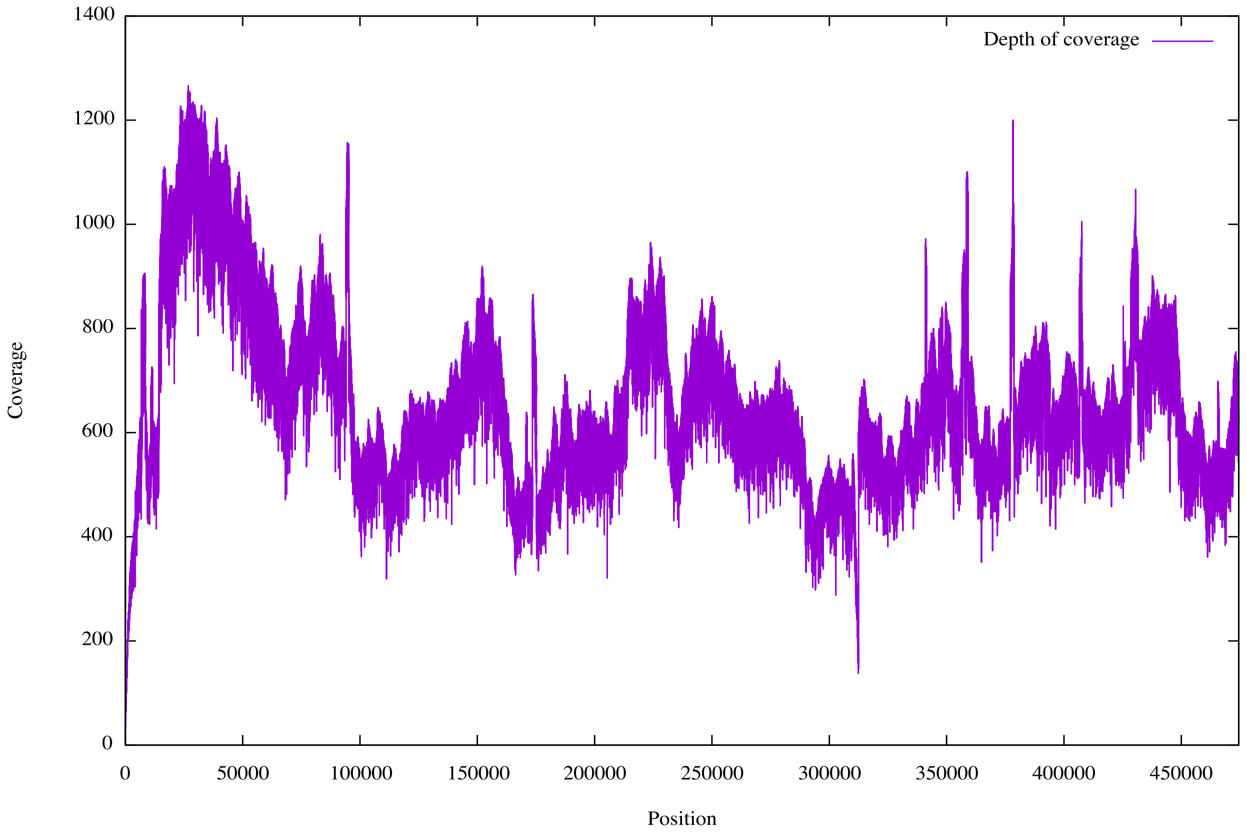

Supplementary Tables

Supplementary Table S1. Size of Cicero (unitig 0 to 6) and Desirée (scfB 0 to 5) assembled contigs.

	**Length**		**length**
**Cicero**	470202	**Désirée**	475441
unitig_0	247659	scfB_5	270266
unitig_4	68611	scfB_1	113097
unitig_5	52463	scfB_4	49282
unitig_6	47394		
unitig_1	46138	scfB_0	42277
unitig_3	7937	scfB_3	519

Supplementary Table S2. List of repeated sequences larger than 100 bp and their coordinates. Their orientation is given (+ or -). Molecule 1 = MN114537, molecule 2 = MN114538, molecule 3 = MN114539.

name	start	stop	Size	strand	molecule
R1A	107206	119120	11915	+	MN114537
R1B	294952	306866	11915	-	MN114537
R2A	158335	165835	7501	+	MN114537
R2B	207504	215004	7501	+	MN114537
R3A	1	4396	4396	+	MN114537
R3B	229572	233967	4396	+	MN114537
R4A	346706	348294	1589	+	MN114538
R4B	392401	393988	1588	+	MN114538
R5A	14271	15479	1209	+	MN114537
R5B	311287	312494	1208	+	MN114537
R6A	96960	97478	519	+	MN114537
R6B	250635	251153	519	-	MN114537
R7A	243234	243569	336	+	MN114537
R7B	435878	436213	336	+	MN114539
R8A	152129	152296	168	+	MN114537
R8B	315357	315524	168	+	MN114538
R9A	32982	33146	165	+	MN114537
R9B	365852	366016	165	+	MN114538
R10A	15672	15832	161	+	MN114537
R10B	368097	368257	161	+	MN114538
R11A	12960	13115	156	+	MN114537
R11B	163119	163274	156	-	MN114537
R11C	212288	212443	156	-	MN114537
R12A	33001	33156	156	+	MN114537
R12B	413674	413829	156	+	MN114538
R13A	16933	17068	136	+	MN114537
R13B	327067	327202	136	-	MN114538
R14A	234321	234448	128	+	MN114537
R14B	366904	367031	128	-	MN114538
R15A	313181	313304	124	+	MN114538
R15B	380232	380355	124	-	MN114538
R16A	243025	243147	123	+	MN114537
R16B	324124	324246	123	+	MN114538
R17A	391739	391852	114	+	MN114538
R17B	445381	445494	114	+	MN114539
R18A	391813	391923	111	+	MN114538
R18B	442262	442372	111	+	MN114539
Repeats found in initial contigs, merged after manual assembly					
**name**	**start**	**stop**	**Size**	**strand**	
R1'A	107206	109514	2309	+	MN114537
R1'B	304559	306866	2308	+	MN114537
R1''A	116438	119120	2683	-	MN114537
R1''B	294952	297635	2684	-	MN114537
R2'A	158335	160593	2259	+	MN114537
R2'B	207504	209762	2259	+	MN114537
R2''A	163624	165835	2212	+	MN114537
R2''B	212793	215004	2212	+	MN114537

Supplementary Table S3. Coordinates of oligonucleotides used to confirm by PCR the order of unitigs (Cicero). The orientation in the genome is given (+ or -).

start	stop	strand	oligo name
**Molecule 1 (MN114537)**			
921	942	–	P1 R
4431	4452	–	P2 R
14767	14789	–	P3 R
100982	101003	–	P4 R
107763	107784	–	P5 R
116372	116393	+	P6 F
116701	116722	–	P2 F
119155	119176	–	P6 R
158056	158077	+	P7 F
160545	160567	+	P8 F
160624	160646	–	P7 R
163510	163531	+	P4 F
166007	166029	–	P4 R
207249	207270	+	P11/7 R
209714	209736	+	P8 F
215060	215081	–	P4/8 R
230201	230222	+	P9 F
230492	230513	–	P1 R
233992	234013	–	P9 R
245907	245928	+	P10 R
294659	294680	+	P1 F
297350	297371	+	P2 F
306288	306309	+	P5 F
307474	307495	+	P13 F
307811	307832	–	P5 R
309286	309307	+	P10 F
309357	309378	–	P13 R
310008	310029	+	P3 F
310883	310904	–	P10 R
311783	311805	–	P3 R
**Molecule 2 (MN114538)**			
1783	1803	–	P14 R
34158	34179	+	P15 F
35860	35881	–	P15 R
54890	54912	–	P3 R
73596	73616	+	P18 R
75629	75651	–	P18 F
79730	79752	+	P16 F
81767	81789	–	P16 R
112703	112724	+	P14 F
**Molecule 3 (MN114539)**			
3406	3428	–	P17 R
49106	49128	+	P17 F

Supplementary Table S4. 5' and 3' transcript extremities as evaluated from the coverage of RNAseq data. Orientation was inferred from the orientation of coding sequences and of editing sites. Consesus promoter elements found upstream of mapped 5' extremities or of tRNA genes are underlined.
Molecule 1 = MN114537, molecule 2 = MN114538, molecule 3 = MN114539.

Molecule 1

5'end	Strand	3'end	Strand	putative promoters		Strand
2476	+	4388	+	37732	CTTGAGAAAT**CATA**AGAGAAGAAGAAA	–
3312	+	12403	–	80058	CTAGAAATAT**CATA**AGAGAAGAAAGAA	–
15020	–	15685	–	86911	TCAGGAATTG**CGTA**AGAGAAGAGACGT	+
18378	–	30663	–	89013	GCTTATGTAA**TATATAA**AGAAGAACCCT	+
32763	–	31108	–	97947	AGATTTCTTC**CATA**ATTGAAGGATATT	+
36851	–	33300	–	114022	AAGCAAAAAT**CATA**AGAGAAGAAAAGT	–
37448	–	33388	–	130198	TATATTATCT**TATATAA**GATAAGCGGA	–
37708	–	50358	–	148251	CTAAGAATAT**CGTA**TATAGTGTATATAG	+
55758	–	56998	–	159443	CAAGATATTG**CGTA**TAAAGAGAAAGATA	+
58596	–	60956	–	189541	TGCTTCATTT**CGTA**TAGTGATAACGCT	–
64583	–	64959	–	208611	CAAGATATTG**CGTA**TAAAGAGAAAGATA	+
74278	–	76893	–	260473	TAATATTATT**TATATAA**TAATAATAAGA	+
80043	–	92306	+	300049	AAGCAAAAAT**CATA**AGAGAAGAAAAGTT	+
86928	+	99166	+			
89028	+	108768	–			
97963	+	111723	–			
111839	–	112023	–			
114003	–	121088	+			
117215	+	125528	–			
130183	–	139093	–			
139953	–	150638	+			
148267	+	165798	+			
159460	+	173483	+			
171088	+	214968	+			
208626	+	235101	+			
232043	+	243561	+			
232878	+	246828	+			
242623	+	271718	–			
242641	+	290918	–			
243034	+	302048	+			
245683	+	302349	+			
278865	–	305293	+			
296843	–	310653	–			
300068	+					
300143	+					
302233	+					
311980	–					

Molecule 2

5'end	Strand	3'end	Strand	putative promoters		Strand
9934	–	6096	–	9954	GTCATTCTCT**TATATAG**TGTAGTCCCCA	–
35797	–	14859	+	10520	AAGCGTATGTG**ATATAA**TCATTCTTATC	+
38694	–	33459	–	42503	CAGAATAGCT**TATATAG**TCATGATTCAA	+
42519	+	51201	–	43025	ATAATAATTA**TATATAA**TTATCTAATAA	+
55785	–	71690	–	55794	TTGAATATCT**TATATAA**TTAATATAAGG	–
73830	–	76286	–	79643	AGCCGGGCGA**CATA**AAAACTTTCGTACA	–
79624	–	98016	–	83885	AAAATAATAT**CGTA**TGTTAATAAAAGAC	–

83862	+			84746	GCCTATCTCT**CATA**AGAGAAGGAGAGCC	–
101486	–			101501	TTCGAAATAT**CATA**AGAGAAGAAAGCTG	–

Molecule 3

5'end	Strand	3'end	Strand	putative promoters		Strand
23304	–	16381	–	9808	TTTTAGTAAA**CGTA**TATAAGCAGCTCTT	+
35334	+	36140	+	40875	TGATGATGAGTATATAAATCAATAAA	–
35365	+	38694	+	47088	CAGAGAATTGCGTAGATAGGAGGACG	–
43284	–	41539	–			
45880	–					
47072	–					

Supplementary Table S5. Location and frequency of editing sites. Only sites observed in more than 10% of reads in two of the three (ADE1, ADE3 and ADE4) independent libraries were considered. Sites with less than 90% editing were annotated as partial edited. Molecule 1 = MN114537, molecule 2 = MN114538, molecule 3 = MN114539.

Molecule 1

Gene	position	annotation	strand	freq_ADE1	freq_ADE3	freq_ADE4	Mean
5' UTR	3851	C>U	+	100	100	100	100
nad1e	3874	C>U	+	100	100	100	100
	3880	C>U	+	100	100	100	100
	3883	C>U	+	100	100	100	100
	3895	C>U	+	98	92	100	97
	3919	C>U	+	100	100	100	100
	3963	C>U	+	100	97	93	97
	4038	C>U	+	100	100	100	100
	4049	C>U partial	+	27	19	29	25
	4056	C>U partial	+	32	26	35	31
	4068	C>U	+	100	100	100	100
	4077	C>U	+	100	100	100	100
	4079	C>U partial	+	24	NA	24	24
3' UTR	4138	C>U partial	+	41	42	38	40
	4242	C>U partial	+	21	23	NA	22
	7770	C>U	-	97	83	92	90
	10374	C>U partial	-	NA	75	80	77
atp1	13243	C>U	-	98	99	99	99
	13318	C>U	-	100	100	100	100
	13441	C>U	-	100	100	100	100
	13517	C>U	-	100	100	100	100
	13555	C>U	-	99	100	99	99
	13694	C>U	-	100	99	99	99
	17144	C>U partial	-	22	51	53	39
trnF(GAA)	17615	C>U partial	-	50	67	50	55
mttB	31368	C>U	-	99	99	95	98
	31413	C>U	-	97	94	85	92
	31414	C>U	-	100	94	86	93
	31463	C>U partial	-	17	39	30	27
	31465	C>U	-	100	95	95	97
	31471	C>U	-	100	99	100	100
	31503	C>U	-	98	99	96	98
	31527	C>U	-	100	99	100	100
	31533	C>U	-	100	100	99	100
	31540	C>U	-	100	100	100	100
	31576	C>U	-	100	100	99	100
	31584	C>U	-	98	100	97	98
	31609	C>U	-	100	100	100	100
	31640	C>U partial	-	47	56	48	50
	31644	C>U	-	100	99	98	99
	31674	C>U	-	100	100	100	100
	31702	C>U partial	-	93	80	64	78

	31705	C>U	-	97	99	100	99
	31707	C>U	-	99	98	100	99
	31708	C>U	-	98	96	95	96
	31735	C>U	-	100	100	100	100
	31737	C>U	-	98	94	83	91
	31750	C>U	-	100	98	90	96
	31753	C>U	-	98	94	85	92
	31754	C>U partial	-	90	82	78	83
	31796	C>U partial	-	34	27	24	28
	31819	C>U	-	98	97	98	98
	31829	C>U partial	-	92	88	83	88
	31857	C>U	-	97	96	95	96
	31879	C>U	-	100	100	100	100
	31893	C>U	-	100	99	96	99
	31903	C>U	-	100	100	100	100
	31950	C>U	-	100	97	99	99
	31951	C>U partial	-	58	33	34	40
	31953	C>U	-	100	100	100	100
	31969	C>U partial	-	81	89	88	86
	31981	C>U	-	88	99	100	95
	32017	C>U	-	99	98	99	99
	32022	C>U partial	-	41	28	21	29
	32044	C>U partial	-	76	40	37	49
	32055	C>U	-	98	98	99	99
	32056	C>U partial	-	74	59	57	63
	32065	C>U	-	98	99	100	99
	32100	C>U	-	100	100	100	100
	32125	C>U	-	95	99	100	98
orf265	32264	C>U partial	-	27	33	33	31
	32984	C>U partial	-	NA	35	NA	35
	32995	C>U partial	-	81	50	NA	64
	33012	C>U partial	-	27	30	NA	28
	45053	C>U partial	+	25	20	NA	22
nad2e	50404	C>U	-	100	100	100	100
	50445	C>U	-	99	100	100	99
	50452	C>U	-	100	100	100	100
	50453	C>U	-	100	100	100	100
	50461	C>U	-	100	100	100	100
	50469	C>U partial	-	41	37	49	42
	50563	C>U	-	100	100	100	100
Intron	51922	C>U partial	-	39	24	28	30
nad2dc	52053	C>U	-	100	100	100	100
	52082	C>U	-	99	100	100	100
	52083	C>U	-	100	100	100	100
	52117	C>U partial	-	27	35	16	25
	52129	C>U partial	-	33	31	25	30
	52301	C>U	-	100	100	100	100
	52367	C>U	-	100	100	100	100
	52371	C>U	-	100	100	100	100

	52401	C>U	-	100	100	100	100
	52474	C>U partial	-	89	86	85	87
	52520	C>U	-	100	100	100	100
	52529	C>U	-	100	100	100	100
	52541	C>U	-	100	100	100	100
	55123	C>U	-	100	100	100	100
5' UTR	55369	C>U partial	-	88	60	53	65
	55613	C>U partial	-	82	72	75	76
3' UTR	61236	C>U	-	100	100	99	100
	61425	C>U	-	100	100	98	99
nad5b	61831	C>U	-	100	100	99	100
	62306	C>U	-	100	99	98	99
	62322	C>U	-	98	95	94	96
	62340	C>U partial	-	40	60	60	52
	62416	C>U	-	100	100	100	100
	62428	C>U	-	100	100	100	100
	62512	C>U	-	100	100	100	100
	62533	C>U	-	100	100	100	100
	62543	C>U	-	100	100	100	100
	62602	C>U	-	100	100	83	94
	62743	C>U	-	100	97	100	99
	62767	C>U	-	100	100	100	100
	62782	C>U	-	100	100	100	100
	62783	C>U	-	100	100	100	100
	62899	C>U	-	100	100	100	100
Intron	62915	C>U	-	100	100	100	100
	62920	C>U	-	100	100	100	100
nad5a	63830	C>U	-	96	94	90	93
nad4bcd	65039	C>U	-	100	100	100	100
	65044	C>U	-	100	98	98	98
	65060	C>U	-	100	100	100	100
	65072	C>U	-	100	100	100	100
	65082	C>U	-	100	100	100	100
	67731	C>U	-	100	100	100	100
	67749	C>U	-	100	100	100	100
	67797	C>U	-	100	99	100	100
	67932	C>U	-	100	100	100	100
	67952	C>U partial	-	18	16	12	15
	67953	C>U	-	100	100	100	100
	67956	C>U	-	100	100	100	100
	67975	C>U	-	100	100	100	100
	67995	C>U	-	100	97	98	99
	68088	C>U	-	100	100	99	100
	68094	C>U	-	100	100	99	100
	68098	C>U	-	100	100	98	99
	71259	C>U	-	100	100	100	100
	71283	C>U	-	100	100	100	100
	71348	C>U	-	100	100	100	100
	71398	C>U partial	-	75	77	62	71

	71399	C>U	-	100	100	100	100
	71468	C>U	-	100	100	95	98
	71576	C>U	-	100	100	92	97
	71627	C>U	-	100	100	100	100
	71658	C>U	-	100	100	100	100
Intron	71961	C>U partial	-	33	48	44	41
	72396	C>U partial	-	42	38	NA	40
nad4a	73209	C>U	-	100	92	88	93
	73210	C>U	-	99	100	100	100
	73213	C>U	-	99	100	100	100
	73270	C>U	-	98	96	91	95
	73284	C>U	-	100	92	100	97
	73385	C>U	-	94	85	100	93
	73449	C>U	-	100	100	100	100
	73480	C>U	-	100	100	100	100
	73488	C>U	-	100	100	100	100
	73490	C>U partial	-	100	86	57	79
	73492	C>U	-	99	100	100	100
	73522	C>U partial	-	100	88	71	86
	73539	C>U	+	100	100	100	100
	73569	C>U	-	100	100	100	100
	73572	C>U	-	100	100	100	100
	73617	C>U	-	100	96	100	99
3' UTR	73804	C>U partial	-	NA	73	63	68
orf125	73875	C>U partial	-	12	40	33	25
	75314	C>U partial	-	50	28	22	31
	75323	C>U partial	-	NA	50	43	46
	79282	C>U partial	-	55	40	NA	47
	80248	C>U partial	+	NA	50	60	55
	81569	C>U partial	+	NA	50	42	46
	83593	C>U partial	+	75	60	60	65
5'-UTR	90013	C>U	+	90	98	100	96
	90139	C>U	+	100	95	98	98
rps4	90241	C>U	+	100	100	100	100
	90253	C>U	+	100	100	80	93
	90265	C>U	+	100	100	100	100
	90310	C>U	+	100	100	100	100
	90457	C>U	+	100	88	86	91
	90891	C>U	+	100	100	100	100
	90901	C>U	+	100	100	100	100
	90916	C>U	+	100	98	100	99
	90967	C>U	+	100	100	96	99
	90981	C>U	+	100	90	100	97
5'-UTR	91635	C>U	+	100	100	92	97
nad6	91732	C>U	+	100	100	100	100
	91794	C>U	+	100	100	97	99
	91801	C>U	+	100	100	97	99
	91809	C>U	+	100	100	100	100
	91853	C>U partial	+	56	71	70	65

	91867	C>U	+	100	99	100	100
	91897	C>U	+	100	100	100	100
	91901	C>U partial	+	11	44	56	30
	92066	C>U partial	+	20	38	36	30
	92275	C>U	+	100	100	95	98
	92860	C>U partial	+	40	38	NA	39
	95591	C>U partial	+	44	42	NA	43
nad4L	98034	C>U	+	97	99	100	99
	98067	C>U	+	100	100	100	100
	98073	C>U	+	98	100	100	99
	98081	C>U	+	96	100	100	99
	98112	C>U	+	100	100	100	100
	98121	C>U	+	100	100	100	100
	98126	C>U	+	100	100	100	100
	98136	C>U	+	100	100	100	100
	98157	C>U	+	100	100	100	100
	98184	C>U	+	100	100	100	100
	98205	C>U	+	100	100	100	100
	98214	C>U	+	100	100	100	100
	98223	C>U	+	99	100	100	100
	98278	C>U partial	+	64	70	77	70
	98307	C>U	+	100	100	100	100
atp4	98576	C>U	+	100	100	100	100
	98588	C>U	+	100	100	100	100
	98606	C>U	+	100	100	100	100
	98712	C>U partial	+	12	15	28	17
	98732	C>U	+	100	100	100	100
	98744	C>U	+	100	100	100	100
	98765	C>U	+	99	100	100	100
	98767	C>U partial	+	35	41	38	38
	98768	C>U	+	99	100	100	100
	98912	C>U	+	100	100	100	100
	98924	C>U	+	100	100	100	100
	98933	C>U	+	100	100	100	100
	105599	C>U partial	-	50	43	70	53
	106346	C>U partial	-	29	58	43	42
	112987	mA	-	39	57	73	55
nad1d	118383	C>U	+	NA	89	100	94
	118413	C>U	+	100	89	100	96
matR	119303	C>U partial	+	57	61	51	56
	119345	C>U	+	88	100	81	89
	119346	C>U	+	95	100	95	97
	119436	C>U partial	+	64	81	83	75
	119523	C>U	+	95	90	93	93
	120655	C>U partial	+	43	28	22	30
	120789	C>U	+	100	100	97	99
	120810	C>U	+	99	100	98	99
	120844	C>U	+	98	100	98	99
	120866	C>U	+	100	100	100	100

	120897	C>U	+	100	100	100	100
	120915	C>U partial	+	30	28	NA	29
	120954	C>U	+	100	100	100	100
nad5de	125699	C>U	-	98	100	100	99
	125739	C>U	-	100	100	100	100
	125741	C>U	-	100	99	100	100
	125762	C>U	-	100	99	100	100
	127012	C>U partial	-	75	77	60	70
	127142	C>U	-	100	100	99	100
	127172	C>U	-	100	100	99	100
	127184	C>U	-	100	100	100	100
	127202	C>U	-	100	100	100	100
	127262	C>U	-	100	100	100	100
orf152	127680	C>U partial	-	NA	22	23	22
5' UTR	127926	C>U	-	100	98	98	99
	128089	C>U partial	-	47	45	48	47
	129852	C>U partial	-	67	32	28	39
	132175	C>U partial	-	33	50	NA	41
	132249	C>U partial	-	NA	38	50	44
	132879	C>U partial	+	50	33	40	40
3' UTR	139162	C>U partial	-	40	33	38	37
5' UTR	139764	C>U partial	-	55	44	41	46
	140101	C>U partial	-	60	30	NA	42
	140106	C>U	-	100	100	80	93
	142581	C>U partial	+	100	50	50	63
	146930	C>U partial	+	50	40	100	58
	147818	C>U partial	+	50	33	NA	41
5' UTR	148883	C>U partial	+	83	92	69	81
nad1a	149169	C>U	+	100	100	100	100
	149219	C>U	+	99	100	100	100
	149261	C>U	+	100	100	100	100
	149262	C>U	+	100	100	100	100
	149290	C>U partial	+	19	42	37	31
rps19	159700	C>U	+	100	91	100	97
	159722	C>U partial	+	75	86	100	86
	159747	C>U	+	100	100	94	98
	159748	C>U	+	100	100	94	98
	159805	C>U	+	80	100	100	93
rps3	161043	C>U partial	+	39	87	100	70
	161077	C>U partial	+	27	13	8	14
	161269	C>U partial	+	30	28	24	27
	161463	C>U	+	92	97	100	96
	161664	C>U	+	93	94	95	94
	161982	C>U	+	100	90	100	97
	162315	C>U	+	100	100	94	98
	162342	C>U	+	100	100	98	99
	162506	C>U	+	88	94	91	91
	162539	C>U	+	95	96	94	95
	162570	C>U	+	88	83	100	90

rpl16	162716	C>U partial	+	35	16	16	21
	162718	C>U	+	100	80	100	93
	162782	C>U partial	+	25	19	17	20
	162846	C>U	+	100	100	96	99
	162935	C>U partial	+	86	63	60	69
	162948	C>U partial	+	16	16	13	15
	163021	C>U	+	100	95	100	98
cox2	163322	C>U partial	+	64	79	70	71
	163336	C>U	+	100	100	100	100
	163369	C>U	+	100	100	100	100
	163405	C>U	+	100	100	100	100
	163459	C>U	+	100	100	100	100
	163551	C>U	+	100	100	100	100
	163576	C>U	+	100	100	100	100
	163677	C>U	+	100	100	100	100
	165126	C>U	+	100	100	100	100
	165143	C>U partial	+	53	72	77	67
	165159	C>U	+	100	100	100	100
	165240	C>U	+	100	100	100	100
	165315	C>U partial	+	94	100	67	86
	165404	C>U	+	100	100	100	100
	165425	C>U	+	100	100	100	100
	165836	C>U	+	100	100	100	100
	170685	C>U partial	+	NA	33	100	57
5' UTR	171194	C>U partial	+	45	18	40	32
	172057	C>U partial	+	23	33	38	31
	172341	C>U partial	+	33	24	30	29
	172741	C>U partial	+	19	33	33	28
ccmC	172757	C>U	+	100	100	94	98
	172758	C>U partial	+	69	83	63	71
	172766	C>U	+	82	100	94	92
	172770	C>U partial	+	55	69	63	62
	172774	C>U partial	+	22	67	NA	38
	172836	C>U	+	94	90	91	91
	172844	C>U partial	+	56	37	67	51
	172867	C>U	+	100	100	100	100
	172885	C>U	+	100	100	100	100
	172913	C>U	+	100	100	100	100
	172931	C>U	+	100	100	100	100
	172936	C>U	+	100	100	100	100
	172983	C>U partial	+	39	36	36	37
	173004	C>U partial	+	83	90	85	86
	173033	C>U	+	100	100	100	100
	173051	C>U	+	100	100	100	100
	173083	C>U	+	99	100	100	100
	173151	C>U	+	98	97	96	97
	173152	C>U	+	100	100	100	100
	173170	C>U partial	+	58	33	43	44
	173173	C>U	+	100	100	100	100

	173198	C>U	+	100	100	99	100
	173203	C>U	+	99	100	99	99
	173210	C>U	+	100	100	100	100
	173215	C>U	+	99	100	100	100
	173219	C>U	+	99	99	98	99
	173225	C>U	+	99	99	99	99
	173249	C>U	+	100	100	100	100
	173300	C>U	+	100	100	100	100
	173320	C>U	+	100	100	100	100
	173327	C>U	+	100	100	100	100
	173357	C>U	+	98	95	95	96
	173358	C>U partial	+	80	81	85	82
	173360	C>U	+	100	98	100	99
	173366	C>U	+	100	100	100	100
	173371	C>U	+	100	100	100	100
	173376	C>U	+	96	98	98	97
	173402	C>U	+	100	100	100	100
	173407	C>U partial	+	12	24	25	19
	173408	C>U	+	100	100	100	100
	173425	C>U	+	100	100	100	100
	174318	C>U	-	100	100	100	100
	191302	C>U partial	+	67	50	100	69
rps19	208869	C>U partial	+	78	82	82	81
	208916	C>U	+	86	88	100	91
	208917	C>U	+	86	83	98	89
	208974	C>U partial	+	86	88	80	85
rps3	210212	C>U partial	+	33	87	100	66
	210246	C>U partial	+	24	11	10	14
	210438	C>U partial	+	29	26	23	26
	210632	C>U	+	93	95	91	93
	210833	C>U	+	93	92	99	95
	211151	C>U	+	100	91	100	97
	211484	C>U	+	79	96	95	90
	211511	C>U	+	98	97	97	97
	211675	C>U	+	100	100	91	97
	211708	C>U	+	100	100	93	98
	211739	C>U	+	96	95	95	95
rpl16	211885	C>U partial	+	22	14	11	15
	211887	C>U	+	97	98	95	97
	211951	C>U partial	+	22	19	16	19
	212015	C>U	+	96	94	95	95
	212104	C>U partial	+	46	39	34	39
	212190	C>U	+	99	99	99	99
cox2	212491	C>U partial	+	23	18	16	19
	212505	C>U	+	100	99	99	99
	212538	C>U	+	100	98	98	99
	212574	C>U	+	100	94	95	96
	212609	C>U partial	+	100	71	33	62
	212623	C>U partial	+	29	33	NA	31

	212628	C>U	+	98	100	98	99
	212720	C>U	+	97	98	98	98
	212745	C>U	+	99	99	99	99
	212846	C>U	+	93	89		91
	214295	C>U	+	98	100	95	98
	214312	C>U	+	100	100	100	100
	214328	C>U	+	100	100	99	100
	214409	C>U	+	100	99	100	100
	214484	C>U	+	99	99	100	99
	214573	C>U	+	99	100	100	100
	214594	C>U	+	100	99	98	99
	218197	C>U partial	+	NA	50	60	55
	220969	C>U partial	+	67	50	NA	58
Intron	233422	C>U	+	100	100	99	100
nad1e	233445	C>U	+	100	100	100	100
	233451	C>U	+	100	100	100	100
	233454	C>U	+	100	100	100	100
	233466	C>U	+	97	98	97	97
	233490	C>U	+	97	100	100	99
	233534	C>U	+	100	99	97	99
	233609	C>U	+	100	99	99	99
	233620	C>U partial	+	21	13	26	19
	233627	C>U partial	+	19	21	28	22
	233639	C>U	+	100	100	100	100
	233648	C>U	+	100	100	100	100
3' UTR	233709	C>U partial	+	70	71	87	76
	233813	C>U partial	+	22	21	15	19
atp6	234004	C>U	+	100	100	94	98
	234227	C>U partial	+	63	67	40	55
	234384	C>U	+	93	100	98	97
	234463	C>U	+	100	98	100	99
	234520	C>U	+	100	100	100	100
	234571	C>U	+	100	100	99	100
	234576	C>U	+	100	100	100	100
	234583	C>U	+	96	96	98	97
	234601	C>U	+	100	100	100	100
	234616	C>U	+	100	100	100	100
	234617	C>U partial	+	72	88	75	78
	234807	C>U	+	100	100	99	100
	234832	C>U	+	100	100	100	100
	234874	C>U	+	100	99	100	100
	234935	C>U partial	+	30	55	57	45
	235003	C>U	+	100	100	100	100
	235011	C>U	+	100	100	100	100
	235018	C>U	+	100	100	100	100
	235025	C>U partial	+	21	22	26	23
	235065	C>U	+	100	100	100	100
3' UTR	237614	C>U partial	+	NA	43	50	46
	237687	C>U partial	+	15	55	100	44

orf263	237842	C>U partial	-	66	13	50	35
	242347	C>U partial	+	32	64	86	56
atp9	243165	C>U	+	99	100	100	100
	243195	C>U	+	100	100	100	100
	243226	C>U	+	96	87	88	90
	243227	C>U	+	100	100	100	100
	243235	C>U	+	100	100	98	99
	243237	C>U	+	100	100	100	100
	243327	C>U	+	100	100	100	100
	243336	C>U	+	100	100	100	100
	243357	C>U	+	100	100	100	100
	243368	C>U	+	100	100	100	100
	244932	C>U partial	+	NA	45	40	42
Intron (nad5c)	245973	C>U partial	+	62	43	26	41
	246216	C>U	+	100	100	100	100
	247427	C>U partial	+	33	40	33	35
	249494	C>U partial	+	NA	46	30	37
nad7d	271784	C>U	-	99	100	100	100
	271813	C>U partial	-	79	86	82	82
	271826	C>U	-	100	100	100	100
	271847	C>U	-	100	100	100	100
	271862	C>U	-	100	100	100	100
	271893	C>U	-	100	100	100	100
	271900	C>U	-	100	98	98	99
	271977	C>U	-	100	100	99	100
	271987	C>U partial	-	68	17	18	28
	272006	C>U	-	100	100	99	99
	272024	C>U partial	-	80	48	30	49
Intron	272153	C>U	-	99	100	100	100
nad7c	273921	C>U	-	100	100	99	100
	273950	C>U	-	98	99	100	99
	273951	C>U	-	100	99	100	100
	273966	C>U	-	100	100	100	100
	274112	C>U	-	100	100	100	100
	274246	C>U partial	-	54	32	39	41
	274307	C>U	-	100	100	100	100
	274346	C>U	-	100	100	100	100
	274355	C>U	-	100	100	100	100
	274374	C>U	-	100	100	100	100
	274375	C>U partial	-	24	29	23	25
	274439	C>U	-	100	99	100	100
	274446	C>U	-	100	99	100	100
Intron	274486	C>U	-	100	100	100	100
nad7ab	275939	C>U	-	100	100	100	100
	275948	C>U	-	100	100	100	100
	276980	C>U	-	100	100	100	100
	276986	C>U	-	100	100	100	100
	277018	C>U partial	-	52	56	57	55
	277025	C>U	-	100	100	100	100

	277039	C>U partial	-	24	13	15	17
5' UTR	277447	C>U partial	-	49	42	30	40
3' UTR	291786	C>U	-	96	100	99	98
nad1bc	292275	C>U	-	100	100	90	97
	292330	C>U	-	100	100	100	100
	292373	C>U partial	-	52	64	56	57
	292374	C>U	-	100	100	100	100
	292410	C>U	-	100	100	100	100
	292417	C>U	-	100	100	100	100
	292418	C>U	-	97	98	97	97
	293957	C>U	-	100	100	100	100
3' UTR	294280	C>U partial	-	95	88	78	87
	294291	C>U partial	-	36	27	19	26
	294379	C>U partial	-	79	71	65	71
rps13	294611	C>U	-	95	86	92	91
	294798	C>U	-	100	100	96	99
	294838	C>U	-	100	100	NA	100
	294842	C>U	-	100	100	96	99
nad1d	295659	C>U partial	-	44	41	61	48
Intron	295689	C>U	-	85	90	100	91
rrn18S	301085	mA	+	73	57	39	55
	307020	C>U partial	+	14	50	24	26
orf320	311099	C>U partial	-	NA	31	32	31
	311254	C>U partial	-	NA	26	25	25

Molecule 2

Gene	position	annotation	strand	freq_ADE1	freq_ADE3	freq_ADE4	mean
ccmFCb	6185	C>U partial	-	96	100	60	83
	6214	C>U partial	-	100	88	63	82
	6219	C>U partial	-	100	96	60	83
	6293	C>U	-	100	96	98	98
	6324	C>U partial	-	27	25	24	25
	6517	C>U partial	-	35	23	23	26
Intron	6744	C>U partial	-	84	69	59	70
	7029	C>U partial	-	61	74	71	68
ccmFCa	7695	C>U	-	100	100	100	100
	7696	C>U	-	100	100	100	100
	7996	C>U	-	100	86	100	95
	8011	C>U	-	97	92	100	96
	8060	C>U partial	-	71	65	73	69
	8068	C>U	-	96	100	100	99
	8087	C>U partial	-	38	24	56	37
	8092	C>U	-	100	100	100	100
	8247	C>U	-	100	100	100	100
	8251	C>U	-	98	100	98	99
	8256	C>U	-	100	100	98	99
	8279	C>U partial	-	46	50	29	41
	8280	C>U	-	100	100	100	100
	8299	C>U	-	100	99	92	97

	8315	C>U partial	-	14	19	NA	16
	8350	C>U	-	100	92	100	97
	8352	C>U	-	100	92	100	97
	8364	C>U	-	100	96	100	99
5' UTR	8413	C>U partial	-	68	70	50	62
	8591	C>U partial	-	42	23	12	23
	10823	C>U partial	+	46	80	100	72
	11002	C>U partial	+	13	23	20	18
	11384	C>U	+	85	88	100	91
	11421	C>U partial	+	68	86	60	71
	11484	C>U partial	+	36	82	75	60
	13280	C>U partial	+	78	78	33	59
	13330	C>U partial	+	17	50	40	32
	13622	C>U partial	+	NA	44	50	47
	13918	C>U partial	+	34	54	33	39
	14572	C>U partial	+	28	22	13	20
	14597	C>U partial	+	63	55	75	64
	14652	C>U partial	+	6	44	26	19
Cob	33622	C>U	-	100	100	99	100
	33724	C>U	-	100	100	100	100
	33798	C>U	-	100	100	100	100
	33853	C>U	-	100	100	83	94
	34026	C>U	-	100	100	100	100
	34138	C>U	-	89	100	99	96
	34348	C>U	-	98	99	100	99
	34381	C>U	-	95	95	96	95
	34408	C>U	-	90	99	98	96
	34592	C>U partial	-	28	17	22	22
	34653	C>U partial	-	31	34	33	33
	35760	C>U partial	-	66	65	69	67
3' UTR	51160	C>U partial	-	75	67	100	80
sdh4	51237	C>U	-	100	100	99	100
	51242	C>U	-	96	100	98	98
	51269	C>U partial	-	18	33	20	23
	51277	C>U partial	-	38	55	46	46
	51310	C>U partial	-	20	37	23	26
	51331	C>U	-	100	99	99	99
	51387	C>U	-	100	100	100	100
	51398	C>U	-	100	100	100	100
	51404	C>U partial	-	56	42	46	48
	51435	C>U	-	100	100	100	100
	51437	C>U	-	94	100	100	98
cox3	51553	C>U partial	-	57	66	68	63
	51561	C>U	-	100	100	100	100
	51662	C>U	-	100	100	100	100
	51803	C>U	-	100	90	89	93
	51896	C>U	-	96	96	100	97
	52001	C>U	-	100	100	100	100
	52004	C>U	-	100	100	100	100
	52011	C>U	-	100	100	100	100

	52017	C>U	-	100	100	100	100
	52070	C>U	-	100	100	100	100
3' UTR	52943	C>U	-	96	100	100	99
atp8	52975	C>U	-	94	100	100	98
	53145	C>U partial	-	44	41	32	39
	53253	C>U partial	-	17	38	34	28
	53342	C>U	-	100	100	92	97
	53360	C>U	-	100	100	100	100
	53371	C>U	-	100	100	100	100
	53379	C>U partial	-	93	81	73	82
	53388	C>U	-	93	89	100	94
5' UTR	53528	C>U partial	-	23	41	43	34
	54071	C>U partial	-	26	43	45	37
	54179	C>U partial	-	39	58	43	46
	54209	C>U partial	-	34	64	49	47
orf118	54474	C>U	-	100	100	96	99
5' UTR	54808	C>U partial	-	28	26	18	24
	56350	C>U partial	-	33	80	NA	51
	57204	C>U partial	-	25	25	NA	25
	61809	C>U partial	+	NA	50	50	50
ccmFN	72162	C>U	-	100	100	100	100
	72197	C>U	-	100	100	100	100
	72209	C>U	-	100	100	100	100
	72233	C>U	-	100	100	100	100
	72236	C>U partial	-	NA	31	35	33
	72252	C>U partial	-	12	33	36	24
	72276	C>U	-	100	100	97	99
	72294	C>U	-	100	100	100	100
	72327	C>U	-	99	100	100	100
	72345	C>U	-	100	100	100	100
	72377	C>U	-	100	100	100	100
	72405	C>U	-	100	100	100	100
	72723	C>U	-	96	100	96	97
	72858	C>U partial	-	33	55	46	44
	72872	C>U	-	100	100	100	100
	72887	C>U	-	100	100	96	99
	72899	C>U	-	100	100	100	100
	72925	C>U partial	-	65	50	72	62
	72949	C>U partial	-	63	63	21	43
	72959	C>U	-	96	100	100	99
	72968	C>U	-	100	100	100	100
	72971	C>U partial	-	21	61	50	40
	73053	C>U partial	-	35	34	42	37
	73153	C>U partial	-	70	69	76	72
	73294	C>U	-	100	NA	100	100
	73301	C>U partial	-	82	82	83	82
	73413	C>U	-	100	100	100	100
	73440	C>U	-	100	100	100	100
	73448	C>U	-	100	100	100	100

	73545	C>U	-	100	100	100	100
	73554	C>U	-	100	100	100	100
	73559	C>U	-	100	100	100	100
	73658	C>U	-	88	96	94	93
	76414	C>U	-	100	100	100	100
	76424	C>U	-	100	100	100	100
	76480	C>U	-	98	100	100	99
	76508	C>U	-	100	100	100	100
	76727	C>U	-	100	100	100	100
	77152	C>U	-	100	100	100	100
cox1	77167	C>U	-	100	100	100	100
	77245	C>U	-	100	100	100	100
	77323	C>U	-	100	100	100	100
	77362	C>U	-	100	100	100	100
	77461	C>U	-	100	100	100	100
	77659	C>U partial	-	100	100	67	87
	77671	C>U partial	-	100	100	67	87
	77898	C>U partial	-	87	87	83	86
Intragenic	77902	C>U	-	99	100	100	100
	77911	C>U	-	98	98	94	97
rps10b	78107	C>U	-	100	100	100	100
	78136	C>U	-	100	100	83	94
Intron	78954	C>U partial	-	50	52	48	50
rps10a	78975	C>U	-	97	100	100	99
	79211	C>U	-	100	100	96	99
	80043	C>U	-	100	100	100	100
	80076	C>U	-	100	100	100	100
cob (repeat)	80103	C>U	-	100	100	100	100
	80287	C>U partial	-	26	25	18	23
	80348	C>U partial	-	32	36	33	34
	81454	C>U partial	-	76	73	79	76
rps14*	81776	C>U partial	-	42	44	36	41
	82096	C>U	-	100	100	95	98
	82099	C>U	-	100	93	85	92
	82442	C>U	-	93	100	93	95
rpl5	82510	C>U	-	100	100	100	100
	82538	C>U partial	-	100	100	67	87
	82543	C>U	-	100	100	100	100
	82555	C>U	-	100	100	100	100
	82567	C>U	-	92	100	100	97
	92204	C>U partial	-	NA	88	57	71
	92786	C>U partial	-	33	50	50	44
	98254	C>U	-	89	95	98	94
	98866	C>U partial	-	36	60	67	53
	99599	C>U	-	100	100	100	100
rps12	99672	C>U	-	86	100	100	95
	99722	C>U	-	100	100	100	100
	99764	C>U	-	100	96	89	95
nad3	99924	C>U	-	96	100	100	99

	99929	C>U	-	98	100	100	99
	99956	C>U	-	98	99	100	99
	99991	C>U partial	-	20	17	11	15
	99998	C>U	-	100	100	96	99
	100007	C>U	-	100	100	97	99
	100022	C>U	-	100	98	98	99
	100026	C>U	-	99	100	97	99
	100043	C>U	-	100	100	100	100
	100058	C>U	-	98	100	98	99
	100064	C>U	-	100	100	99	100
	100065	C>U	-	100	100	100	100
	100126	C>U partial	-	52	34	14	29
	100127	C>U	-	99	100	100	100
	100135	C>U partial	-	NA	43	24	32
	100194	C>U partial	-	27	51	41	38
	100211	C>U	-	97	100	100	99
	100212	C>U	-	93	100	92	95
	100229	C>U	-	99	100	100	100
	100230	C>U partial	-	28	18	9	17
	100268	C>U	-	100	99	100	100
5' UTR	100288	C>U partial	-	NA	11	17	13
3' UTR	100393	C>U partial	-	86	88	67	79
orf265	101174	C>U partial	-	14	31	39	26
5' UTR	101371	C>U partial	-	21	41	48	35
	101898	C>U	-	NA	100	100	100
	109789	C>U partial	+	33	20	40	30
	109807	C>U partial	+	33	25	40	32

Molecule 3

Gene	position	annotation	strand	freq_ADE1	freq_ADE3	freq_ADE4	mean
	9972	C>U	+	100	100	100	100
	9983	C>U	+	100	93	100	98
	9987	C>U	+	100	98	100	99
	10024	C>U	+	100	98	100	99
	10031	C>U partial	+	86	78	86	83
	10072	C>U	+	100	100	100	100
	10081	C>U	+	100	100	100	100
	10092	C>U	+	100	100	100	100
	10093	C>U	+	100	97	100	99
9ccmB	10098	C>U	+	100	98	100	99
	10104	C>U	+	100	100	100	100
	10108	C>U	+	100	98	100	99
	10116	C>U	+	100	98	100	99
	10123	C>U	+	100	100	97	99
	10127	C>U partial	+	87	65	54	67
	10137	C>U	+	100	99	100	100
	10138	C>U	+	100	99	100	100
	10230	C>U	+	100	100	100	100
	10239	C>U partial	+	50	46	47	48

	10248	C>U	+	99	100	100	100
	10257	C>U	+	100	100	100	100
	10281	C>U partial	+	82	71	67	73
	10282	C>U	+	100	100	100	100
	10311	C>U	+	99	100	100	100
	10319	C>U partial	+	26	36	41	34
	10324	C>U	+	98	97	99	98
	10368	C>U	+	100	100	100	100
	10372	C>U	+	100	100	100	100
	10411	C>U	+	99	99	100	99
	10419	C>U	+	100	100	100	100
	10420	C>U	+	100	100	100	100
	10424	C>U	+	100	100	100	100
	10429	C>U	+	99	100	100	100
	10438	C>U	+	99	100	100	100
	10446	C>U partial	+	89	71	77	79
	10447	C>U	+	99	100	98	99
	10456	C>U	+	99	98	99	99
	10458	C>U	+	99	99	99	99
	10495	C>U	+	100	100	99	100
	10498	C>U	+	100	99	100	100
	10510	C>U	+	100	100	100	100
	10513	C>U	+	100	99	100	100
	10516	C>U	+	100	95	99	98
	10520	C>U	+	99	94	96	96
	10540	C>U	+	100	100	100	100
	10555	C>U	+	99	100	100	100
3' UTR	10571	C>U partial	+	36	33	32	34
	10582	C>U partial	+	34	64	57	50
atp8 (repeat)	10680	C>U	+	100	100	100	100
	10689	C>U	+	100	100	100	100
	10710	C>U	+	100	100	100	100
	10721	C>U	+	100	100	100	100
3' UTR	16389	C>U	+	100	98	100	99
rps10	16681	C>U	-	100	97	100	99
	16786	C>U	-	98	100	99	99
	16819	C>U	-	100	100	100	100
Intron	17625	C>U partial	-	29	32	33	31
rpl2	19909	C>U	-	100	100	100	100
orf210	20818	C>U partial	-	24	51	43	37
	26606	C>U partial	-	100	50	25	50
	34227	C>U partial	+	33	75	NA	50
	34906	C>U partial	+	NA	50	67	58
	35288	C>U partial	+	50	24	25	31
sdh3	35473	C>U	+	98	100	100	99
	35603	C>U partial	+	17	37	27	26
3'-UTR	35767	C>U partial	+	35	59	50	47
	35769	C>U partial	+	43	53	50	48
	35770	C>U	+	100	100	100	100

	35779	C>U partial	+	76	84	75	78
nad2a	36327	C>U	+	87	93	97	92
Intron	37410	C>U partial	+	71	81	89	80
nad2b	37540	C>U	+	100	100	97	99
	37569	C>U partial	+	93	83	66	80
	37625	C>U	+	99	98	93	97
	37658	C>U	+	100	100	100	100
	37673	C>U	+	100	100	100	100
	37678	C>U	+	100	100	100	100
	37684	C>U	+	100	100	100	100
	37718	C>U	+	100	100	100	100
	37745	C>U	+	100	100	100	100
	37814	C>U	+	98	100	100	99
	37840	C>U	+	100	100	100	100
Intron	37870	C>U partial	+	40	47	53	46
nad9	41723	C>U	-	97	95	100	97
	41764	C>U	-	100	100	100	100
	41794	C>U	-	99	95	95	96
	41834	C>U	-	100	100	100	100
	41864	C>U	-	100	100	100	100
	41995	C>U	-	100	100	99	100
	42070	C>U	-	100	100	100	100
5' UTR	42165	C>U partial	-	27	40	37	34
	42248	C>U	-	95	91	86	91
	42690	C>U partial	-	57	75	64	65
	43541	C>U partial	-	17	22	30	22
	44431	C>U partial	-	18	46	60	37

Supplementary Table S6. List of regions with high similarity (BLAST hits) resulting form pairwise comparisons between the potato mitogenome and mitochondrial genome of Solanaceae species available in GenBank. BLAST hits were filtered based on the size of the alignment ≥ 5kb and on sequence identity ≥ 95% and represent syntenic blocks. The list of mitochondrial genes within each syntenic block is also reported. Molecule 1 = MN114537, molecule 2 = MN114538, molecule 3 = MN114539.

Query id[a]	Subject id	% Identity	Alignment length	Mismatches	Gap openings	Q. start	Q. end	S. start	S. end	Bit score	mtgenes[b]
MF989960.1	MN114537	99,91	52552	28	8	136244	188790	310730	258191	1,04E+05	rrn5, rrn18, rps13, **nad1bc**, nad7,**trnI**, **trnM**
MF989960.1	MN114537	99,9	49490	27	5	1	49480	184572	234050	9,77E+04	trnM, trnG, trnQ, rps19, rps3, rpl16, cox2, nad1e
MF989960.1	MN114538	99,9	37338	30	4	72589	109925	2854	40185	7,37E+04	ccmFC, trnN, cob, trnS
MF989960.1	MN114537	99,82	29506	29	6	281013	310512	136694	107206	5,81E+04	nad5de, matR, **nad1d**, rrn18, rrn5
MF989960.1	MN114539	99,6	28298	56	12	210868	239145	20969	49229	5,52E+04	trnE, sdh3, nad2ab, trnW, trnP, nad9, trnH
MF989960.1	MN114537	99,95	15780	7	1	298597	314376	294952	310730	3,12E+04	**nad1d,** rrn18, rrn5
MF989960.1	MN114537	99,95	11916	5	1	140108	152023	107206	119120	2,36E+04	rrn5, rrn18, **nad1d**
MF989960.1	MN114539	99,5	10934	40	5	239146	250076	1	10922	2,12E+04	trnK, ccmB
MF989960.1	MN114538	99,92	10385	7	1	266744	277128	68972	79355	2,05E+04	ccmFN, cox1, rps10
MF989960.1	MN114538	99,81	9844	15	1	109877	119716	40207	50050	1,94E+04	trnD, trnS
MF989960.1	MN114537	99,92	9267	3	1	191574	200840	234307	243569	1,83E+04	partial atp6, atp9

MF989960.1	MN114538	99,68	9075	18	4	119652	128725	50047	59111	1,77E+04	sdh4, cox3, atp8
MF989960.1	MN114537	99,66	9062	19	4	257683	266740	6815	15868	1,77E+04	atp1
MF989960.1	MN114537	99,84	7551	8	1	330877	338427	177025	184571	1,49E+04	
MF989960.1	MN114537	99,96	7504	2	1	22933	30436	158335	165837	1,48E+04	rps19, rps3, rpl16, cox2
MF989960.1	MN114537	99,93	7388	1	1	249741	257128	243234	250617	1,46E+04	nad5c
MF989960.1	MN114537	99,95	6619	3	0	66177	72795	145708	152326	1,31E+04	**nad1a**
MF989960.1	MN114539	99,88	6421	8	0	200505	206925	10587	17007	1,27E+04	rpl10
MF989961.1	MN114537	99,93	58335	28	6	1	58325	15481	73810	1,15E+05	trnP, trnF,trnS,**mttB,** rrn26,trnfM, nad2cde, trnY, trnN, trnC, nad5ab, nad4
MF989961.1	MN114537	99,91	48688	26	8	87432	136114	306866	258191	9,61E+04	rrn5, rrn18, **nad1d**, matR,rps13, nad1bc, nad7, **trnI**, **trnM**
MF989961.1	MN114537	99,93	41026	28	2	58323	99347	78096	119120	8,11E+04	rps4, nad6, nad4L, atp4, rrn5, rrn18, **nad1d**
MF989961.1	MN114539	99,6	28298	56	12	158192	186469	20969	49229	5,52E+04	trnE, sdh3, **nad2ab**, trnW, trnP, nad9, trnH
MF989961.1	MN114539	99,5	10934	40	5	186470	197400	1	10922	2,12E+04	trnK, ccmB
MF989961.1	MN114537	99,92	9267	3	1	138898	148164	234307	243569	1,83E+04	partial atp6, atp9
MF989961.1	MN114537	99,67	8674	17	4	205007	213676	6815	15480	1,70E+04	atp1

MF989961.1	MN114537	99,93	7388	1	1	197065	204452	243234	250617	1,46E+04	nad5c
MF989961.1	MN114539	99,88	6421	8	0	147829	154249	10587	17007	1,27E+04	rpl10

NC_035963.1	MN114537	99.78	57837	97	6	138766	196577	64647	6815	1.137e+05	atp1, trnP, trnF, trnS,mttB,rrn26, trnM, **nad2cde,** trnY, trnN, trnC,**nad5ab**
NC_035963.1	MN114537	99.73	25324	61	4	257724	283045	107213	132531	4.964e+04	rrn5, rrn18, **nad1d**, matR,**nad5de**
NC_035963.1	MN114537	99.77	20023	23	8	338794	358812	257618	277620	3.929e+04	trnI,nad7
NC_035963.1	MN114537	99.74	16152	40	2	375183	391334	294444	310593	3.167e+04	rps13,**nad1d,**rrn18,rrn5
NC_035963.1	MN114537	99.75	15644	37	2	253989	269632	310593	294952	3.069e+04	rrn5, rrn18, **nad1d,**
NC_035963.1	MN114538	99.55	15579	45	8	28510	44072	96645	81076	3.030e+04	rps14*,rpl5
NC_035963.1	MN114537	99.76	14623	26	3	290384	305002	92713	78096	2.870e+04	nad6,rps4
NC_035963.1	MN114537	99.64	14294	31	6	358837	373120	277612	291894	2.791e+04	
NC_035963.1	MN114538	99.62	14307	28	8	69101	83385	40185	25884	2.790e+04	trnS, cob, trnN
NC_035963.1	MN114538	99.76	13977	32	2	91589	105564	16891	2916	2.742e+04	ccmFc
NC_035963.1	MN114537	99.60	13548	32	5	432220	445759	228159	214626	2.642e+04	
NC_035963.1	MN114538	99.80	13406	26	1	313945	327350	79355	65951	2.635e+04	rps10,cox1,ccmFN

NC_035963.1	MN114539	99.63	13384	27	3	210437	223816	46583	33218	2.616e+04	trnH,nad9,trnP,trnW,**nad2ab**,sdh3
NC_035963.1	MN114537	99.83	12727	17	4	112831	125556	214621	201898	2.503e+04	**cox2**,rpl16,rps3,rps19, trnQ
NC_035963.1	MN114537	99.83	12727	17	4	257	12982	214621	201898	2.503e+04	**cox2**,rpl16,rps3,rps19,trnQ,
NC_035963.1	MN114537	99.71	11909	33	1	375691	387599	119120	107213	2.333e+04	**nad1d**, rrn18, rrn5
NC_035963.1	MN114537	99.36	9895	31	7	128522	138403	198865	188990	1.911e+04	trnM
NC_035963.1	MN114537	99.36	9895	31	7	15948	25829	198865	188990	1.911e+04	trnM
NC_035963.1	MN114537	99.88	8909	9	2	305000	313907	73810	64903	1.756e+04	nad4
NC_035963.1	MN114539	99.71	8961	21	1	399954	408914	20200	11245	1.756e+04	rpl2,rpl10
NC_035963.1	MN114539	99.71	8961	21	1	236409	245369	11245	20200	1.756e+04	rpl10,rpl2
NC_035963.1	MN114538	99.72	8222	13	3	83376	91596	25330	17118	1.611e+04	
NC_035963.1	MN114537	99.59	7397	16	3	197132	204527	250617	243234	1.442e+04	**partial atp9, nad5c**
NC_035963.1	MN114537	99.92	7119	5	1	112831	119949	165452	158335	1.406e+04	**cox2**,rpl16,rps3,rps19
NC_035963.1	MN114537	99.92	7119	5	1	257	7375	165452	158335	1.406e+04	**cox2**,rpl16,rps3,rps19
NC_035963.1	MN114538	99.54	6792	21	3	60285	67072	49802	43017	1.321e+04	trnS, trnD
NC_035963.1	MN114537	99.79	6707	6	1	330448	337146	152822	146116	1.319e+04	
NC_035963.1	MN114537	99.18	5698	27	6	44251	49948	103622	97945	1.090e+04	atp4, nad4L

NC_035963.1	MN114538	98.48	5519	57	8	54565	60077	55544	50047	1.025e+04	rps1, atp8, cox3,sdh4
NC_035964.1	MN114537	99.84	47721	59	3	93783	141488	64649	16933	9.399e+04	nad5ab,trnC,trnN, trnY,nad2cde, trnM,rrn26,mttB,trnS, trnF, trnP
NC_035964.1	MN114537	99.75	25324	56	4	191834	217155	107213	132531	4.968e+04	rrn5,rrn18, matR, **nad5de**
NC_035964.1	MN114537	99.77	20023	23	8	32172	52190	257618	277620	3.929e+04	trnI,nad7
NC_035964.1	MN114537	99.75	18439	44	3	66488	84926	292158	310593	3.616e+04	**nad1bc,**rps13,**nad1d**, rrn18, rrn5
NC_035964.1	MN114538	99.59	16747	44	8	160194	176923	79907	96645	3.262e+04	cob*, rps14*, rpl5
NC_035964.1	MN114537	99.77	15644	34	2	188099	203742	310593	294952	3.071e+04	rrn5, rnn18,**nad1d**
NC_035964.1	MN114537	99.77	14623	25	3	224494	239112	92713	78096	2.871e+04	nad6, rps4,
NC_035964.1	MN114537	99.66	14294	31	5	52215	66502	277612	291894	2.794e+04	
NC_035964.1	MN114537	99.65	13548	30	4	294782	308325	228159	214626	2.647e+04	partial cox2
NC_035964.1	MN114538	99.82	13406	23	1	7389	20794	79355	65951	2.638e+04	rps10, cox1,ccmFN
NC_035964.1	MN114539	99.66	13384	23	3	378260	391639	33218	46583	2.619e+04	sdh3,**nad2ab**, trnW, trnP, nad9,trnH
NC_035964.1	MN114537	99.83	12727	16	4	308855	321579	214621	201898	2.503e+04	cox2, rpl16, rps3, rps19, trnQ
NC_035964.1	MN114537	99.74	11909	30	1	69283	81191	119120	107213	2.335e+04	**nad1d**,rrn18, rrn5
NC_035964.1	MN114539	99.80	10923	16	3	394310	405227	1	10922	2.146e+04	trnK, ccmB

NC_035964.1	MN114538	99.75	9918	16	3	151874	161783	25884	35800	1.945e+04	trnN,cob
NC_035964.1	MN114537	99.38	9895	29	7	324545	334426	198865	188990	1.913e+04	trnM
NC_035964.1	MN114537	99.67	9051	25	1	412842	421887	6815	15865	1.770e+04	atp1
NC_035964.1	MN114537	99.88	8909	9	2	239110	248017	73810	64903	1.756e+04	nad4
NC_035964.1	MN114539	99.71	8961	21	1	248055	257015	20200	11245	1.756e+04	rpl2,rpl10
NC_035964.1	MN114538	99.85	8837	13	0	258167	267003	11752	2916	1.742e+04	ccmFc
NC_035964.1	MN114538	99.74	8222	11	3	143663	151883	17118	25330	1.612e+04	
NC_035964.1	MN114537	99.59	7397	16	3	404892	412287	243234	250617	1.442e+04	**partial atp9, nad5c**
NC_035964.1	MN114537	99.92	7119	5	1	308855	315973	165452	158335	1.406e+04	cox2,rpl16, rps3, rps19
NC_035964.1	MN114538	99.59	6792	18	3	350010	356797	43017	49802	1.323e+04	**trnD**,trnS
NC_035964.1	MN114537	99.81	6707	5	1	23892	30590	152822	146116	1.320e+04	**nad1a**
NC_035964.1	MN114537	99.93	6050	3	1	274282	280331	165452	159404	1.195e+04	**cox2,**rpl16, rps3,rps19
NC_035964.1	MN114537	99.93	6050	3	1	274282	280331	214621	208573	1.195e+04	**cox2**,rpl16, rps3,rps19
NC_035964.1	MN114537	99.45	5686	23	4	337328	343013	97945	103622	1.100e+04	nad4L, atp4

KJ865410.1	MN114537	99,06	13867	78	15	361940	375757	64067	50205	2,64E+07	nad5ab,trnC,trnN, trnY,nad2cde

KJ865410.1	MN114538	98,59	9472	76	12	313872	323298	14655	5197	1,74E+04	ccmFC
KJ865410.1	MN114538	98,83	7585	54	9	164256	171816	68530	76103	1,43E+07	ccmFN
KJ865410.1	MN114537	99,29	7465	32	12	83029	90480	66272	73728	1,39E+04	partial nad4
KJ865410.1	MN114537	98,81	6478	36	10	1764	8206	277173	270702	1,20E+04	nad7
KJ865410.1	MN114537	97,89	6347	84	12	102623	108944	121911	128232	1,15E+07	nad5de
KJ865410.1	MN114539	98,13	5826	56	11	343393	349179	46117	40306	1,03E+04	trnH,nad9,trnP, trnW
KJ865410.1	MN114537	98,43	5174	36	8	54099	59228	43004	37832	9418	
KJ865410.1	MN114537	98,92	5093	26	9	494579	499658	159405	164481	9228	rps19,rps3,rpl16, cox2 ex2
KJ865410.1	MN114537	98,92	5093	26	9	494579	499658	208574	213650	9228	rps19,rps3,rpl16, cox2 ex2

NC_006581.1	MN114537	98,85	10098	72	11	372284	382356	268731	278809	1,87E+04	nad7
NC_006581.1	MN114538	98,9	8462	76	6	301817	310272	13641	5191	1,56E+04	ccmFC
NC_006581.1	MN114537	99,2	8158	34	11	182205	190352	73810	65674	1,56E+07	nad4
NC_006581.1	MN114537	99,15	7041	50	3	344384	351420	25137	32171	1,33E+04	mttB
NC_006581.1	MN114537	98,16	6647	90	7	550	7181	124571	117942	1,20E+04	**nad1d**, matR
NC_006581.1	MN114537	98,77	5922	36	8	192100	197997	64033	58125	1,08E+04	nad5ab,trnC

NC_006581.1	MN114539	98,41	5159	50	6	274123	279273	40285	35151	9,58E+03	nad2ab
NC_029805.1	MN114537	98,86	10098	71	11	372284	382356	268731	278809	1,87E+04	nad7
NC_029805.1	MN114538	98,91	8462	75	6	301817	310272	13641	5191	1,56E+04	ccmFC
NC_029805.1	MN114537	99,22	8157	34	10	182206	190352	73810	65674	1,56E+07	nad4
NC_029805.1	MN114537	99,15	7041	50	3	344384	351420	25137	32171	1,33E+04	mttB
NC_029805.1	MN114537	98,18	6647	89	7	550	7181	124571	117942	1,22E+07	**nad1d**, matR
NC_029805.1	MN114537	98,77	5922	36	8	192100	197997	64033	58125	1,08E+04	nad5ab,trnC
NC_029805.1	MN114539	98,41	5159	50	6	274123	279273	40285	35151	9,58E+03	nad2ab, sdh3
NC_026515.1	MN114537	97,85	7402	92	14	139478	146857	118223	125585	1,31E+04	nad1d,matR
NC_026515.1	MN114537	96,15	7043	88	30	30486	37463	265005	258081	1,19E+07	trnI
NC_026515.1	MN114537	97,68	5293	84	9	167794	173057	146155	151437	9127	nad1a

[a]MF989960.1, MF989961.1 = *S.commersonii*; NC_035963.1 = *S. lycopersicum*; NC_035964.1= *S. pennellii*; KJ865410.1= *C. annuum*; NC_006581.1= *N. tabacum*; NC_029805.1= *N. sylvestris*; NC_026515.1= *H. niger*

[b]In red are indicated genes not annotated in query sequence, in blue genes present in query sequence
